# Historical Routes for Diversification of Domesticated Chickpea Inferred from Landrace Genomics

**DOI:** 10.1093/molbev/msad110

**Published:** 2023-05-09

**Authors:** Anna A Igolkina, Nina V Noujdina, Margarita Vishnyakova, Travis Longcore, Eric von Wettberg, Sergey V Nuzhdin, Maria G Samsonova

**Affiliations:** Mathematical Biology and Bioinformatics Laboratory, Peter the Great St. Petersburg Polytechnic University, St. Petersburg, Russia; Marine and Environmental Biology, University of Southern California, Los Angeles, CA, USA; N.I. Vavilov All-Russian Institute of Plant Genetic Resources (VIR), St. Petersburg, Russia; Spatial Sciences Institute, University of Southern California, Los Angeles, CA, USA; Mathematical Biology and Bioinformatics Laboratory, Peter the Great St. Petersburg Polytechnic University, St. Petersburg, Russia; Plant and Soil Science and Gund Institute for the Environment, University of Vermont, Burlington, VT, USA; Molecular and Computational Biology, University of Southern California, Los Angeles, CA, USA; Mathematical Biology and Bioinformatics Laboratory, Peter the Great St. Petersburg Polytechnic University, St. Petersburg, Russia

**Keywords:** chickpea, admixture graph, allele frequency, compositional data, domestication

## Abstract

According to archaeological records, chickpea (*Cicer arietinum*) was first domesticated in the Fertile Crescent about 10,000 years BP. Its subsequent diversification in Middle East, South Asia, Ethiopia, and the Western Mediterranean, however, remains obscure and cannot be resolved using only archeological and historical evidence. Moreover, chickpea has two market types: “desi” and “kabuli,” for which the geographic origin is a matter of debate.

To decipher chickpea history, we took the genetic data from 421 chickpea landraces unaffected by the green revolution and tested complex historical hypotheses of chickpea migration and admixture on two hierarchical spatial levels: within and between major regions of cultivation.

For chickpea migration within regions, we developed popdisp, a Bayesian model of population dispersal from a regional representative center toward the sampling sites that considers geographical proximities between sites. This method confirmed that chickpea spreads within each geographical region along optimal geographical routes rather than by simple diffusion and estimated representative allele frequencies for each region. For chickpea migration between regions, we developed another model, migadmi, that takes allele frequencies of populations and evaluates multiple and nested admixture events. Applying this model to desi populations, we found both Indian and Middle Eastern traces in Ethiopian chickpea, suggesting the presence of a seaway from South Asia to Ethiopia. As for the origin of kabuli chickpeas, we found significant evidence for its origin from Turkey rather than Central Asia.

## Introduction

Crop domestication is a unique form of biological coevolution with humans to establish new varieties with improved and beneficial phenotypes. Research in domestication is motivated not only by its economic and cultural importance for humans but also by solving fundamental questions, which remain an area of heated debate (Meyer and Purugganan 2013; Stetter et al. 2017): For some species, there is still no consensus on different aspects of domestication, including estimates of the timing, origin, human role, and adaptation during postdomestication divergence and spread.

Geographical origins of domestication of cultivated crop plants were first systematically described by Nikolai Vavilov (Vavilov 1926, 1951). Together with primary centers of origin (where crop wild relatives were first domesticated), Vavilov suggested secondary centers, distinct areas where crops were independently cultivated gaining unique traits and diversity (supplementary fig. S1, Supplementary Material online). Populations in these centers share the evolutionary history of migrations and admixtures. Conventional methods to estimate genetically distinct founder populations are STRUCTURE and ADMIXTURE (Alexander et al. 2009; Pritchard et al. 2000). Along with the calculation of the summarized admixture statistics, there are approaches to estimate local admixtures along chromosomes using kernel smoothing in local principal component analysis (PCA) spaces (Santos et al. 2019) or the “coancestry matrix” (combination of PCA and STRUCTURE information) as in ChromoPainter and fastGLOBETROTTER (Lawson et al. 2012; Wangkumhang et al. 2022). These approaches could predict ancestral populations and major gene flows, but not the history of admixture events. Existing tools to infer the admixture graph for a set of populations—TreeMix (Pickrell and Pritchard 2012) and MixMapper (Lipson et al. 2013)—could handle admixtures with two source populations and/or two nested admixtures. The qpGraph tool from the ADMIXTOOLS2 package can handle a higher number of nested events and mixtures of two sources; however, it operates on a defined topology and does not infer and screen admixture graphs (https://github.com/uqrmaie1/admixtools). However, the story of the domestication of some crops goes beyond these limitations and requires the search in the admixture graph space, and new methods are needed to decipher more complex situations.

**Fig. 1.**
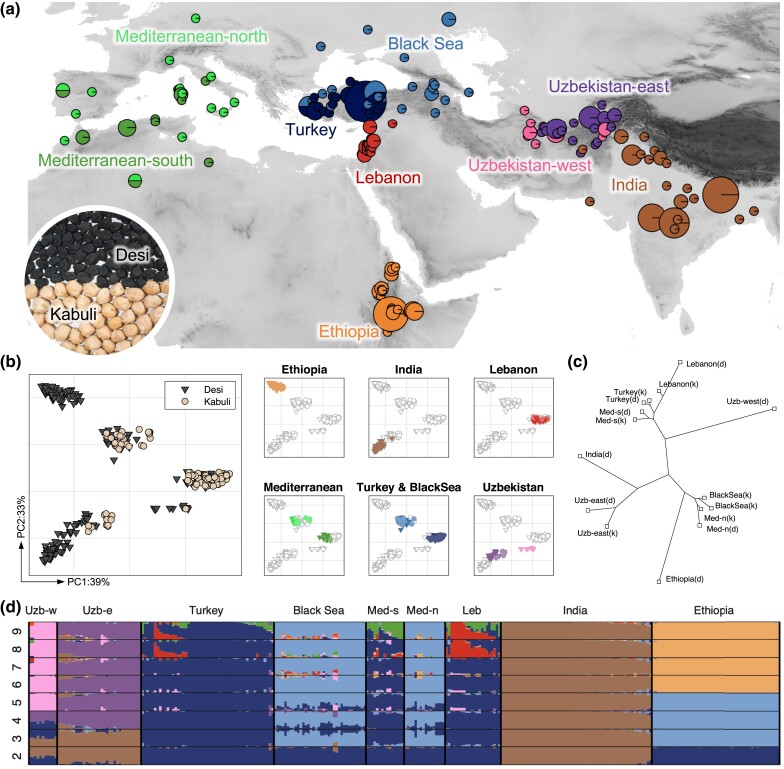
(*a*) Sampling sites of population after filtration. The circle size is proportional to the number of sampled accessions. Two-colored pie charts denote locations in which samples belonging to two different populations have been collected. The photo shows the morphological differences between seeds of desi and kabuli chickpea types (Photo: M. Vishnyakova). (*b*) PCA plots based on SNP data. Accessions are colored with respect to chickpea market type (left) and regions (right). (*c*) Neighbor-joining tree based on mean pairwise *F*_ST_ comparison of chickpea populations. (*d*) Population structure inferred using ADMIXTURE analysis. ADMIXTURE results at *K* = 2..9 are shown. Each accession is represented by a horizontal stacked bar indicating the proportions of ancestry in *K*-predicted ancestral populations.

One of the species with complex and partially obscure migration history is chickpea (*Cicer arietinum* L.), a legume that serves as an essential source of high-quality protein (Abbo et al. 2003a), ranked third among legumes in terms of grain production (Jain et al. 2013). Based on the archeological evidence, the distribution of compatible wild relatives, and pan-genome analysis, the upper reaches of Mesopotamia in Southeastern Turkey is generally accepted as the origin of chickpea (Abbo et al. 2003b; van der Maesen 1987; von Wettberg et al. 2018; Varshney et al. 2021). The domesticated chickpea varieties further spread from the Fertile Crescent westward and eastward into Europe, Northern Africa, and Asia (Varshney et al. 2021). In these new areas, the subsequent diversification of domesticated populations happened through adaptation to different agroecological environments and cultural practices. Recent genetic clustering of chickpea (Varshney et al. 2021) revealed five centers of chickpea diversity in the Old World: the Mediterranean, Central Asia (Uzbekistan), Near East (Turkey and the Black Sea), South Asia (India), and East Africa (Ethiopia). These centers have archaeological records, which partially uncover possible scenarios of chickpea domestication: the spread throughout the ancient world to western–Central Asia and the Indus Valley ca 6,000 ybp, the Mediterranean basin (Lebanon and Morocco) ca 5,500 ybp, and Ethiopia ca 3,500 ybp (Zohary and Hopf 2000; Redden and Berger 2007). However, the exact dispersal and admixture history of chickpea within the Mediterranean Basin and to Ethiopia presents a puzzle. The chickpea's history gets even more complicated due to the presence of two distinct types: “desi” and “kabuli” (fig. 1*a*), which differ in color and morphology (Moreno and Cubero 1978; Purushothaman et al. 2014), although there is no crossing boundary or substantial molecular genetic differentiation between subtypes (Varma Penmetsa et al. 2016). The desi type is considered ancestral and resembles wild progenitors (*Сicer reticulatum* and *Cicer echinospermum*), and kabuli was likely once selected from the local desis and then spread; however, the region of kabuli's origin is not known.

We utilized the genotyped landraces from Vavilov's collection at the N.I. Vavilov All Russian Institute of Plant Genetic Resources (VIR) to test the abovementioned ambiguities in chickpea's history and reconstruct migration routes of both desi and kabuli types. This historical germplasm collection contains landraces, which were collected in the 1920s–1930s, that is, before the Green Revolution, and possibly underwent less intensification of artificial selection than currently cultivated market varieties. Within the collection, we assembled a panel of 421 samples with geographical origins covering all centers of diversification and genotyped at 2,759 loci (Sokolkova et al. 2020). In the data set, we identified 10 chickpea populations (6 desis and 4 kabulis) based on the regional attributions and characterized them with representative allele frequencies.

The ambiguities in chickpea history cannot be resolved with conventional TreeMix (Pickrell and Pritchard 2012) and MixMapper (Lipson et al. 2013) methods, and tools that could handle complex admixture hypothesis with multiple sources and nested admixture events are required. Indeed, it is possible to trace back Ethiopian chickpeas to ancestral populations from Turkey, Lebanon, or India and in each region kabulis could have mixed with local desis. Here, we developed a new method, “migadmi” (migrations and admixtures), which copes with a high number of source populations and multiple nested admixtures. In addition, this tool considers the irregularity of admixture traces along the genome, which can be essential if the admixture event happened far in the past. To estimate representative allele frequencies, we developed the “popdisp” model (population dispersals), which considers geographical locations of chickpea sampling sites, the nonequal number of accessions in sites, and, most crucially, possible ways of chickpea dispersals within a region. After that, we examined hypothetical migrations and complex admixtures between populations.

## Results

### Population Structure

PCA analysis of chickpea genotypes (landraces) demonstrated that the first principal component (PC) mostly reflects the difference between desi and kabuli subtypes; however, the separation is not strict, evincing the absence of any reproductive isolation between the subtypes (fig. 1*b*). The first two PCs explain 72% of variance, and the first 6 PCs explain 90% of variance (supplementary fig. S2*c*, Supplementary Material online).

To detect the underlying population structure, we used ADMIXTURE (Alexander et al. 2009). We ran this tool with a different number of hypothetical founder populations (*K*), but the cross-validation error monotonically decreased with no minimum while increasing K from 1 to 20 (supplementary fig. S2*d*, Supplementary Material online). The ADMIXTURE analysis with *K* = 2 separates Asian regions from the rest, analysis with *K* = 3 distinguishes Ethiopian region together with part of Middle East and Mediterranean samples, analysis with *K* = 4 splits Central Asian and Indian regions as distinct groups, analysis with *K* = 5 divides Central Asian region into two groups, and analysis with *K* = 6 completely separates the Ethiopian cluster and makes the admixture panel conformed to the PCA plot (fig. 1*c*). The six groups of samples obtained were almost isolated geographically; hence, we defined one-to-one correspondence between geographic regions (some called by the closest modern country) and genetic groups: Ethiopian, Indian, Uzbek-east, Uzbek-west, Mediterranean-and-Middle-East-north, and Mediterranean-and-Middle-East-south.

The compact location of admixture groups on the geography supports the presence of the strong geographical signal in genetic data. To reinforce this correspondence, we eliminate possible minor independent gene flows between regions or traces of contamination. Based on the ADMIXRURE analysis with six source populations, we filtered out samples with a contribution from any source population <60%. We considered such samples as highly mixed and not representative of any source population.

Samples with disagreement between the genetic group and the geographic region were filtered out too. As a result, we worked with 294 samples; the ADMIXTURE analysis and geographical locations of the samples before filtration are shown in supplementary figures S3 and S4, Supplementary Material online.

Samples of two admixture groups (Mediterranean-and-Middle-East-north and Mediterranean-and-Middle-East-south) were split further into Mediterranean and Middle East, respectively. The Middle-East-north group was mostly located around the Black sea, so we called it the Black Sea population. Hierarchical clustering of the landraces based on SNP distance showed (supplementary fig. S2*a*, Supplementary Material online) that samples from the Lebanon territory (both desi and kabuli) form a separate clade. Therefore, we subdivided the Middle-East-south population into the so-called Turkey and Lebanese populations. In the result, we distinguished five populations in the Mediterranean-and-Middle-East region: Black Sea, Turkey, Lebanese, Mediterranean-north, and Mediterranean-south.

As the desi/kabuli signal was substantial in the data set, we further split accessions within each region into two groups; there are no kabulis among Ethiopian, Indian, and Uzbek-west landraces in our collection (fig. 1*a*).

To check the genetic separation between populations, we performed the neighbor-joining clustering of them based on *F*_ST_ statistics (fig. 1*d*). The similarity between desi and kabuli subpopulations within each population was higher than the similarity between different populations, which supports their separation. The PCA, ADMIXTURE, and *F*_ST_ results are in line with the previous study (Varshney et al. 2019) that also revealed Fertile Crescent, South Asia, Central Asia, East Africa, and Mediterranean geographical groups, as well as desi/kabuli differentiation patterns among cultivated genotypes. However, these observations are not enough to decipher the migration and domestication history of chickpea accessions.

### Chickpea Dispersals within Geographic Regions

**Fig. 2.**
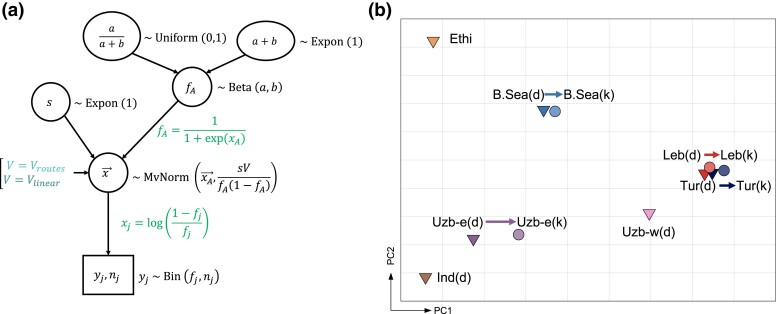
(*a*) popdisp, the hierarchical Bayesian model describes the spread of a population within each region. We consider that a region consists of *J* sampling sites. *j*-th site is characterized with $y_{j}$ allele counts in $n_{j}$ genotyped variants; $y_{j}$ and $n_{j}$ are known values. We assume that $y_{j}$ is a result of binomial sampling with $n_{j}$ trials and $f_{j}$ probability of success (the allele frequency in the site). Allele frequencies, as fractions or percentages, are constrained (i.e., sum up to 1 or 100%), which requires the transformation of all $f_{j}$ into $x_{j}$ being in line with BEDASSLE (Bradburd et al. 2013) and CoDA (Aitchison 1986; Pawlowsky-Glahn and Buccianti 2011). The vector $\vec{x}$ follows the multivariate normal distribution: Its mean is the transformed allele frequency in the center, $x_{A}$, and the covariance matrix is proportional to the covariance matrix *V*. We tested different paths: constructed under “routes” and “linear” hypotheses. We assumed that the values in matrix *V* are precalculated, so the way of estimating the distances between samples does not affect the number of parameters in the model. Allele frequency in the center has the beta prior distribution with *α* and *β* parameters; *s* is the constant of proportionality. (*b*) PCA plot of allele frequencies estimated under the “routes” hypothesis. Arrows represent the shift from desi to kabuli populations within one region. Darker colors represent desi (*d*), and lighter colors represent kabuli (*k*). Shapes also reflect chickpea subtypes: triangle, desi, and circle, kabuli.

Before testing migrations and admixtures for chickpea desi and kabuli populations, we estimated representative allele frequencies for each population. Due to the nonuniform distribution of sampling sites in regions and nonequal number of genotypes in each site (from 2 to 60), mean allele frequencies in each population can be biased as the mean is sensitive to outliers. To get more robust estimates, we developed a model, “popdisp” (fig. 2*a*), which considers a scenario for dispersals within a geographic region and takes into account landrace-specific effects. The Bayesian hierarchical structure of the model was inspired by BayPass (Gautier 2015), and the processing of allele frequencies was performed as in BEDASSLE (Bradburd et al. 2013) and compositional data analysis (CoDA) (Pawlowsky-Glahn and Buccianti 2011).

We considered two scenarios for subsequent dispersal within the region. In the first scenario, dispersal within each region was sensible to the geographic landscape. As a result, the genetic relatedness in local landraces would be predicted by the geographic least-cost paths. This scenario was contrasted with simple diffusion so that genetic differences between landraces would be explained by geodesic distance. We called these two scenarios “routes” and “linear,” respectively (fig. 2*a*).

Proceeding from the idea that the exact site from which chickpea dispersal began in a region is unknown and could be at any location, we inscribed the sampling sites into a rectangle, set back the 0.1 of side lengths from the boundaries, and defined 16 locations on a 4 × 4 even grid. Thus, in each region, we tested 16 origins instead of taking the 1 chosen as, for example, the ancient trade center. For each origin in the region, we considered two scenarios of dispersals (routes and linear) and took frequencies with the highest Bayesian information criterion (BIC) as the representative frequencies for the region.

Sampling sites in the Mediterranean region are located on islands or around the sea; therefore, many of the least-cost paths between them pass over the water. The balance between movement costs on water and on land is challenging; hence, creating the distance matrix between samples in the Mediterranean region is difficult. Therefore, we decided to exclude populations from this region from our analysis. In contrast to the Mediterranean region, samples in the Black Sea population could be connected by the land paths. As a result, we worked with 11 populations: desi and kabuli in Turkey, Lebanese, Black Sea, and Uzbek-east regions and only desi (kabuli was not presented) in Indian, Ethiopian, and Uzbek-west regions.

We separately estimated allele frequencies in 11 populations under the routes and linear scenarios and discriminated between them by BIC values. In almost all cases (except the Ethiopia desi population), the route scenario was strongly favored (supplementary file S1, Supplementary Material online). Therefore, we concluded that the dispersal within regions occurred in agreement with geographic paths and barriers and took allele frequency estimates based on this model for further analysis. PCA analysis of the obtained frequencies demonstrated both splittings of populations into geographic subgroups and desi/kabuli differentiation (fig. 2*b*). Moreover, all kabuli populations are close to their regional desis, but shifted in one direction along the first PC axis.

### Origin of Desi Landraces in Ethiopia

Two alternative hypotheses exist about the chickpea colonization of Ethiopia. On the one hand, the spread of chickpea is inextricably linked with human history, and the origin of Ethiopian chickpeas can be proposed from the ethnic composition of Ethiopians. A haplotype sharing analysis points on Eurasian sources (such as Anatolian or Levant Neolithic ones) of contemporary Ethiopians (Kivisild et al. 2004; Pagani et al. 2012), and Ethiopian highlanders have a clear Semitic connection exemplified by their Semitic language group (Amharic) and genetic similarity with Jewish people (Behar et al. 2010). Besides, human genomic studies revealed backflows into East Africa from West Eurasia around 4,500 years ago (Llorente et al. 2015). Interestingly, archaeological evidence dates the arrival of Near Eastern founder crops into Ethiopia to the same time period (Harrower et al. 2010; Mitchell and Lane 2013). Based on this, chickpea in Ethiopia could have a Middle Eastern origin. On the other hand, Ethiopian landraces are smaller-seeded and dark-colored, like most Indian varieties. Thus, the genome of Ethiopian varieties could be admixed with alleles traced back to ancestral populations from West Asia (modern Turkey and Lebanon) or India.

**Fig. 3.**
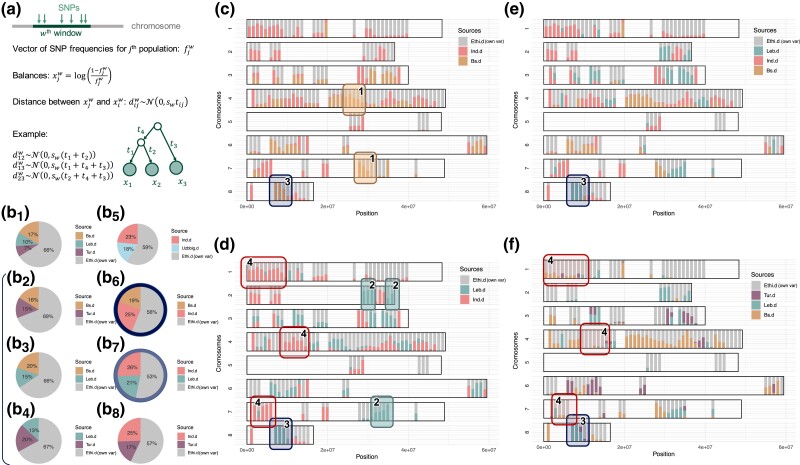
Ethiopian desi chickpeas as an admixed population. (*a*) Parametrization of an admixture event in the migadmi model. First, we split each chromosome in a sliding window technique; each *w*-th window is a set of SNPs. Instead of vectors of SNP frequencies for populations $f_{j}^{w}$, we use vectors of balances $x_{j}^{w}$. We assumed that the distance between vectors of balances within a window follows the normal distributions with covariance proportional to the corresponding admixture tree's distance. (*b*_1–8_) Decomposition of variance of the Ethiopian desi by sources. The highest BIC value corresponds to the decomposition into Black Sea desi (Bs.d) and Indian desi (Ind.d) (circled with the dark blue color). The next likely decomposition is with Lebanese (Leb.d) and Indian desis (circled with light blue color). The vertical bracket shows all pair combinations of Middle East desi populations as sources for the Ethiopian desi. (*c*) Distribution of the contribution of Black Sea (yellow) and Indian (red) ancestral desi populations into Ethiopian desi along chromosomes. Each vertical bar corresponds to the composition estimates in one window; color defines the proportion of admixture from an ancestral population. (*d*) Distribution of contribution of Lebanese (green) and Indian (purple) desi populations into Ethiopian desi along chromosomes. (*e*) Distribution of contribution of Lebanese (green), Indian (red), and Black Sea (yellow) desi populations into Ethiopian desi along chromosomes. (*f*) Distribution of contribution of Lebanese (green), Turkey (purple), and Black Sea (yellow) desi populations into Ethiopian desi along chromosomes. Numbers next to squares in (*c*–*f*) indicate zones to compare between subfigures. Grey color in pie charts and in chromosomal views reflects own dispersion/variation of admixed populations (marked also with “own var”) acquired after mixing event.

With the migadmi model (fig. 3*a*), we tested all possible origins of the Ethiopian desi population, considering it as a mixture of two or three sources. As source populations, we used all desi populations (Turkey, Black Sea, India, Uzbekistan-east, and Lebanon) with the known and fixed topology of phylogeny (supplementary fig. S5, Supplementary Material online). First, we tested all Middle East populations in pairs (fig. 3*b*_2–4_), and the Black Sea population demonstrated the highest contributions to Ethiopian desi. This is consistent with the hypothetical path of chickpeas to Ethiopia from a recent study (Varshney et al. 2019).

At the same time, the Ethiopian population demonstrated a substantial affinity to Asian chickpea varieties: The cumulative impact of Indian and Uzbek-east desi is even higher than the cumulative impact from the Middle East populations (fig. 3*b*_1_ and 3*b*_5_). The highest BIC value among all pairs of desi populations corresponds to the decomposition of Ethiopian desis into the Black Sea and Indian sources (fig. 3*b*_6_), the second highest to the (India + Lebanon) decomposition (fig. 3*b*_7_). When we tested the Ethiopian population as an admixture with three sources, the BIC value didn’t decrease comparing to the (India + Black Sea) admixture, so the Black Sea and Indian desi populations are the main contributors to Ethiopian desis. However, more than half of Ethiopian desi's variance is not represented in ancestral populations, which is in line with the previous analysis, where Ethiopia represents a distinct cluster (fig. 1*b*–*d*).

We compared the (India + Black Sea) (fig. 3*c*) and (India + Lebanon) (fig. 3*d*) admixtures in chromosome-wide plots and found that some chromosomal regions come either only from the Black Sea population (fig. 3*c*, pointer 1) or only from the Lebanese population (fig. 3*d*, pointer 2). However, most of the regions assigned to the Black Sea population in the (India + Black Sea) admixture test are classified as a Lebanese contribution in the evaluation of India and Lebanon contributions (fig. 3*d*–*f*, pointer 3), which means that these regions have no specificity to a particular population, but rather ancestrally belong to the Middle East branch. Chromosome decomposition also shows that some blocks in Ethiopian population have only Indian ancestry (fig. 3*d* and *f*, pointer 4): They were estimated as of Indian origin when Indian desi participated in the analysis and, as own variance, when only Middle East desis were considered as the origin (fig. 3*d*).

TreeMix results indicated Turkey and India as the origins of Ethiopian desi, while MixMapper suggests that Ethiopian desi is a mixture of desi from Black Sea desi (74%) and India (26%) (supplementary text S5, Supplementary Material online). The find_graph() method from the admixtools revealed that the most probable admixture in six desi populations is Ethiopian population, which is the mixture of Black Sea population and India population, which is consistent with TreeMix, MixMapper, and migadmi results. Despite the general agreement of migadmi predictions with TreeMix and MixMapper, we believe that this newly introduced method provides a more realistic picture of chickpea colonization in Ethiopia as it takes into account the accumulation of individual variances in both mixed and source populations after the admixture event and is able to decompose the variance of mixed population along the chromosomes. Indeed, our analysis demonstrated that the nonuniformity of admixture events along chromosomes is strongly pronounced—some regions are admixed by only one source population, while other regions have input from several ones.

### Origin of Desi Landraces in West Uzbekistan

**Fig. 4.**
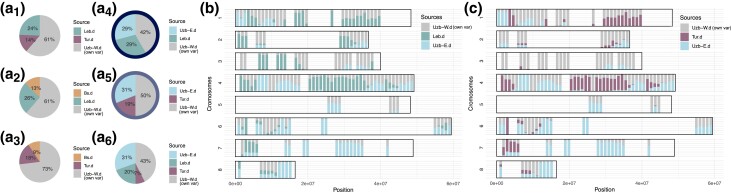
Uzbek-west desi chickpeas as an admixed population. (*a*_1–6_) Decomposition of variance of Uzbek-west desi by sources. The highest BIC value corresponds to the decomposition into Lebanese desi and Uzbek-west desi (circled with the dark blue color). The next likely decomposition is with Turkey and Uzbek-west desis (circled with light blue color). (*b*) Distribution of contribution of Lebanese (green) and Indian (blue) desi populations into Ethiopian desi along chromosomes. Each vertical bar corresponds to the composition estimates in one window; color defines the proportion of admixture from an ancestral population. (*c*) Distribution of contribution of Turkey (purple) and Indian (blue) desi populations into Ethiopian desi along chromosomes.

The Uzbek-west population demonstrates a distinct admixture group and a separate branch on the neighbor-joining tree (fig. 1*d*). We tested it as an admixture of all pairs of the five source desi populations. Among Asian populations, the Uzbek-east desi population had the dominant effect, while among Middle Eastern populations, the input of the Lebanese desis prevails (fig. 4*a*_1–3_). The highest BIC value among all pairs of source populations corresponds to ancestries from Uzbek-east and Lebanese populations (fig. 4*a*_4_, marked with the dark blue circle), and the second highest BIC is pointed to admixture from Uzbek-east and Turkey (fig. 4*a*_5_, marked with the light blue circle). At the chromosomal level, all the blocks with ancestries from Turkey coincide with blocks inferred as originating from the Lebanese population; however, there is a number of blocks with ancestry specifically assigned to the Lebanese population (fig. 4*b* and *c*, third chromosome).

TreeMix, MixMapper, and axmixtools predicted that Uzbek-west desis have ancestry from the Fertile Crescent populations: TreeMix traced ancestry to the Turkish–Lebanese clade, Mixmapper suggested Lebanese ancestry (68%; supplementary text S5, Supplementary Material online), and find_graph() from axmixtools predicted form 50% to 60% Lebanese ancestry of Uzbek-west desis. However, all methods inferred Indian admixture, which due to large geographic distances looks less likely than the Uzbek-west admixture, identified with migadmi.

### Origin of Kabuli Chickpea

Based on linguistic evidence, one may hypothesize that kabulis arose in Central Asia and are named after Kabul city (in modern Afghanistan). On the other hand, the West Asian (modern Turkey) domestication of kabulis (after desis) is also possible, as kabulis are distributed in regions neighboring to Turkey and have long been thought to be modern introductions to India and Ethiopia (van der Maesen 1984). Although desis and kabulis have much in common, breeding programs generally keep them separate, likely due to differences in adaptive requirements and market preferences (Purushothaman et al. 2014; Roorkiwal et al. 2014; Varshney et al. 2019).

**Fig. 5.**
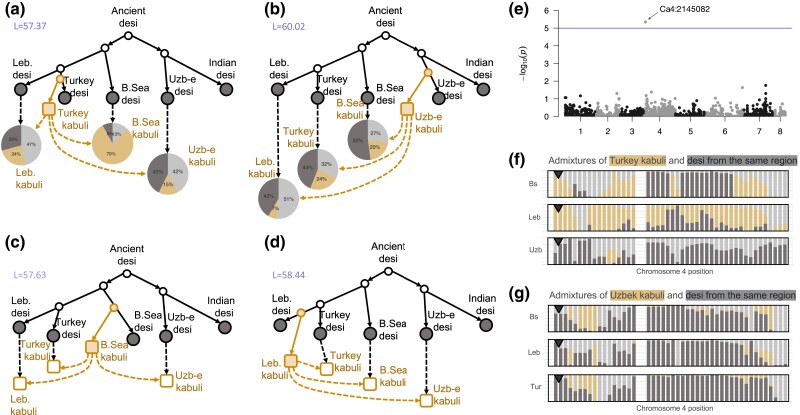
Analysis of the origin of kabuli chickpeas. (*a*) Admixture tree for kabulis assuming that they originated in Turkey. The pie plots reflect (from left to right) the decompositions of Lebanese, Black Sea (B.Sea), and Uzbek-east kabulis variances. Pie diagrams show the decomposition of admixed populations into sources with the corresponding colors (yellow or dark grey); light grey color means the own variance of the population. *L* value means the score of the model: the lower—the better. (*b*) Admixture tree for kabulis assuming that they originated in Uzbekistan (Kabul), Uzbek-east population. The pie plots reflect (from left to right) the decompositions of the Lebanese, Turkish, and Black Sea kabuli variances. (*c*) Admixture tree for kabulis assuming they originated from the Lebanese desi population. (*d*) Admixture tree for kabulis assuming they originated from the Black Sea desi population. (*e*) Manhattan plot for GWAS of the desi/kabuli binary trait (seed color). (*f*) Decomposition of the Black Sea, Lebanese, and Uzbek-east kabuli ancestries along the fourth chromosome under the assumption of Turkish origin of kabulis. Each vertical bar corresponds to the composition estimates in one window; color defines the proportion of admixture from an ancestral population. Triangles mark the chromosomal region associated with desi-kabuli grouping. (*g*) Decomposition of the Black Sea, Lebanese, and Turkish kabuli ancestries along the fourth chromosome under the assumption of the Uzbek-east origin of kabulis. Triangles mark chromosomal regions associated with desi-kabuli grouping.

Thus, four hypotheses were examined in this study: Three of them assumed the dispersal of kabuli chickpea from Turkey's Fertile Crescent, Turkey, Black Sea, or Lebanon specifically (fig. 5*а*, *c* and *d*), and the fourth hypothesis reflected its Central Asian origin (modern Uzbekistan) with a subsequent move back to the Middle East (fig. 5*b*). The BIC value for the fourth model was the lowest, while the model with the highest BIC value predicted the Turkish origin of kabulis; the second highest BIC value, the Lebanese origin of kabulis; the third highest BIC value, the Black Sea origin; and the lowest BIC value, the Uzbek-east origin of kabulis.

Under the Central Asian assumption of kabuli origin, the influence of Uzbek-east kabuli on other kabulis is smaller than under the Turkey origin hypothesis (pie plots in fig. 5*a* and *b*). The analysis of the PCA plot (fig. 2*b*) demonstrated the shift of all kabuli populations along the first PC axis and the direction of this shift is not “toward Uzbekistan.” TreeMix analysis did not reveal significant patterns of kabuli admixture, while MixMapper indicated the same pattern as we found (supplementary text S5, Supplementary Material online). Admixtools analysis of F4 statistics (qpdstat function) and optimal admixture graphs (find_graph function) showed that Uzbek-east kabuli are rather the result of admixture with the Turkey origin (supplementary text S5, Supplementary Material online). Overall, we do not observe support for a kabuli origin in Central Asia with introgression back to Fertile Crescent populations, and we thus cautiously conclude that kabuli originated in the Turkish region.

The most pronounced difference between the desi and kabuli chickpea subtypes is the seed color. In legumes, this trait is Mendelian and controlled by the so-called A gene (Hellens et al. 2010). For *Pisum sativum* and *Medicago truncatula*, the sequences of this gene can be found at GenBank accessions: GU132940 (MtbHLH) and GU132941 (PsbHLH). We took these sequences, performed the TBlastN search against *C. arietinum* genes, and found the match with basic helix–loop–helix protein A located at the LOC101506726 gene (2,149,255–2,158,629 bp, the beginning of chromosome 4). We performed genome-wide association studies (GWAS) for the desi–kabuli contrast using rrBLUP and found one significant SNP, which is located 4,173 bp upstream of LOC101506726 (fig. 5*e*). Therefore, in line with previous findings (Varma Penmetsa et al. 2016) we consider а region around the LOC101506726 gene as a marker for chickpea seed color. Analysis of local admixtures in Lebanese and Black Sea kabuli populations under competing hypotheses (fig. 5*f* and *g*) showed that this region is inherited from Turkish kabulis and not from Uzbek-east kabulis (marked with the triangle in fig. 5*f* and *g*).

## Discussion

We have analyzed chickpea migration and admixture hypotheses directly by testing various dispersal scenarios partially based on historical evidence. We observed that the Ethiopian desi population was derived not solely from the Fertile Crescent, but almost equally from India and the Middle East (Black Sea–Lebanon).

While the BIC value supports the Ethiopian desi as the admixture of two populations from India and the Black Sea region, on a fine scale, at the chromosomal level, we found regions that are specific for Lebanese impact. Therefore, we conclude that the Ethiopian population is a mosaic of ancestry from Indian, Lebanese, and Black Sea source populations. Likewise, a chromosomal admixture pattern for west Uzbek desis (Varshney et al. 2019) has been clarified into two likely land routes of migration: from the Fertile Crescent (Lebanon) and from east Uzbekistan.

Another question we addressed was the origin of kabuli, the light-colored chickpea type, which presumably originated from a local desi population. According to the analysis we performed, this region is Turkey. In line with Varshney et al. (2021), we observed no evidence for kabuli's Central Asia origin and spreading back to the Fertile Crescent. However, our results do not support the parallel migration of chickpeas into India and Ethiopia as was proposed in that work.

To test the migration and admixture hypotheses, we developed two methods. The first model is “popdisp,” which estimates the representative allele frequencies in a population under the assumption of a type of spreading within the region. The spreading type should be provided to the popdisp as the covariance matrix between sampling sites. In our study, for each region, we assumed the spread from a representative center, and the center was used to get the representative allele frequencies of a region. We also suggested the heuristic to estimate covariance matrixes for this spread type. To avoid subjectivity, within each region, we tested 16 locations (on 4 × 4 even grid) as candidates for the center and took allele frequency estimates from the model with the highest BIC value. A user can analyze simpler (i.e., binary tree paths) or more complex spread models if they can be translated into a covariance matrix.

We considered two reasonable migration paths within a region: diffusion with and without considering the geographical landscape. Our analyses unambiguously favor the former scenario: The genetic relatedness between accessions was predicted by the geographic least-cost paths. The least-coast paths minimize the human movement cost between two given locations on a real landscape and could reflect human trade routes. In the future, it will be interesting to apply this approach to species with different dispersal strategies, for instance, comparing crops like round-seeded chickpea to human-associated weeds like spiky-podded *Medicago* capable of long-distance transport with livestock or wind dispersed species. For the latter, we would expect distributions to track wind currents only, with no resulting signature of dispersal along historic trade routes.

The second model is “migadmi,” which estimates multiple and nested admixture hypotheses with more than two sources and demonstrates the admixture patterns along the chromosomes. Both models describe changes in allele frequencies in line with Wright–Fisher drift model and utilize logit transformation as in BEDASSLE (Bradburd et al. 2013) and CoDA, the most appropriate framework for working with frequencies, fractions, percentages, and ratios. This approach allows one to easily extend migadmi and popdisp to work with not only biallelic SNPs but also with multiallelic sites or haploblocks.

## Materials and Methods

### Data set

The chickpea data set (*C. arietinum* L.) consists of 421 accessions from the VIR seed bank. These accessions were genotyped by sequencing (GBS), and 56,855 segregating single nucleotide polymorphisms (SNPs) were identified. These SNPs were further filtered to meet requirements for minor allele frequency (MAF) >3% and genotype call-rate >90%. A total of 2,579 SNPs in 421 accessions passed all filtering criteria and were retained for further analysis (Sokolkova et al. 2020). When accessions were attributed to 6 geographical regions, we picked 10 chickpea populations for the analysis (table 1). Samples in each population were characterized by the same set of SNPs.

**Table 1.** Distribution of the Number of Accessions in Populations. Ethiopian and Indian Regions Contained a Few Numbers of Kabulis. Therefore, for Our Study, We Considered Populations Which Are Highlighted with Green.

	Before filtration			After filtration		
Region	Desi	Kabuli	Other	Desi	Kabuli	Unused
Ethiopia	61	3	0	50	0	14
India	64	5	1	57	0	32
Lebanon	18	16	0	10	12	12
Turkey	40	73	4	13	39	29
Black Sea				18	18	
Uzbekistan-west	70	25	2	10	0	54
Uzbekistan-east				20	13	
Mediterranean-north	19	19	1	5	11	8
Mediterranean-south				9	6	

### Spatial Data and Distance Calculations

For each accession, the exact geographic sites of the sample site are known; some accessions were collected from the same site. Based on the genetic similarity and geographical proximity of samples, we defined nine geographic regions. Within each region, we estimated the location of the chickpea diffusion center in the following manner. We inscribed the sampling sites into a rectangle, set back 0.1 of side lengths from the boundaries, and defined 16 locations on a 4 × 4 even grid (supplementary file S2, Supplementary Material online). Using the popdisp model, we tested each of these locations as a diffusion center and took one with the highest BIC value. In our case, the comparison of models based on BIC values is the same as on likelihood values, because the distance matrix between samples is not parametrized and does not affect the number of parameters in the model.

We considered two diffusion models of chickpea spread from the centers toward sampling sites: linear distances and cost route distances. For the later scenario, we estimated the least-cost paths between all sampling sites and potential centers. To simulate human movement across a landscape, we used the Herzog cost function (leastcostpath R package (Lewis 2021)).

The least-cost path is the explanatory framework for the movement of goods in archeology (Douglas 1994). This approach calculates the least “cost” distance of a path, which can be interpreted as the amount of time or energy that it would have taken to travel along the path. This approach is useful in the absence of historical data on exact movement routes, and it takes into account the change in elevation, the hiking function (Herzog function), geo-climatic Holocene data, and a mask of water bodies.

**Fig. 6.**
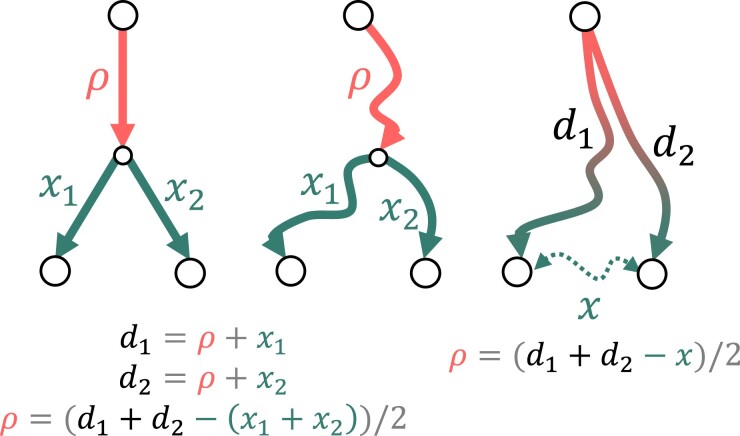
Heuristics for the covariance value between two sampling sites using the pairwise geographical distances between them and the center. ρ, the covariance value; $x_{1}$ and $x_{2}$, distances on the binary tree from the last common node to sampling sites; $d_{1}$, distance between the center and the first sampling site; $d_{2}$, distance between the center and the second sampling site; and *x*, geographical distance between sampling sites.

Based on the distances between sampling sites and the center, we estimated the joint variability (covariance) between sites. If a binary tree presents the spread paths from the center toward sampling sites, the covariance between two sites (ρ) is proportional to their common path on the tree (fig. 6, left). This value can be also expressed as the linear combination of three distances: from each site to the center ($d_{1}$ and $d_{2}$) and between sites (*x*) (formula on fig. 6). Building the optimal tree-like path from the center to the sites is very time-consuming because it requires all points on the surface to be tried and tested as fork points (fig. 6, center). Instead, we used this formula as a heuristic for the more general case when the specific path is unknown (fig. 6, right). We applied it to obtain two covariance matrices based on linear distances and cost route distances.

### Model for Diversification within Clusters

The model describing population dispersals is implemented in Python package “popdisp” (https://github.com/iganna/popdisp). Testing of popdisp on simulated data is provided in the supplementary text S6, Supplementary Material online.

#### Model

We developed popdisp, a Bayesian hierarchical model (fig. 2*a*), that describes the historical diversification of a chickpea population within a geographic region. This model is not specific for chickpea; however, we describe it for this species. We hypothesize that each geographic region contains *M* sites, each characterized by one landrace subpopulation and one center (ancestral site), where chickpea was first introduced and spread from. Each site is characterized by individuals genotyped for *N* unlinked (independent) biallelic SNPs; the missing data are possible and do not require imputation. Assumption of independence means that we can apply the popdisp analysis for each SNP separately. For each biallelic SNP, we randomly assign reference and alternative states, and the popdisp method is insensitive for this assignment. We pooled the data from all individuals in a site, so that, for *j*-th site and *i*-th SNP, we defined the total counts of alternative allele, $y_{j}^{i}$, and the total count of all variants at this SNP, $n_{j}^{i}$. Values $n_{j}^{i}$ are not the same across all SNPs in *j*-th site due to the missing data. We assume that the frequency of the alternative allele for *i*-th SNP in *j*-th site is $f_{j}^{i}$ and the observed $y_{j}^{i}$ follows the binomial distribution: $y_{j}^{i}∼Bin(\;f_{j}^{i},n_{j}^{i}).$

Within a region, we modelled the population spread from the ancestral site (center), which is characterized by respective frequency $f_{A}^{i}$. We assumed that allele frequencies change under the genetic drift in line with the Wright–Fisher model and theory of CoDA. The CoDA theory states that frequencies (as well as percentages or fractions) are meaningless when considered alone, as they sum up to one; hence, the only balances between frequencies do make sense. According to the CoDA, we applied the isometric log-ratio (ilr) transformation to allele frequencies, and, in case of biallelic SNPs, it is the logit transformation as used in BEDASSLE (Bradburd et al. 2013):


$$x_{j}^{i}=\log\frac{1-f_{j}^{i}}{\;f_{j}^{i}};f_{j}^{i}=\frac{1}{1+\exp\;(x_{j}^{i})}.$$

New variable $x_{j}^{i}$ means the log-balance between frequencies of reference and alternative alleles and is not bounded, that is, can take values in (−∞,+∞). The latter allows us to model correlations between allele frequencies using multivariate normal distributions without artificial truncation, which is necessary when the model operates with nontransformed frequencies (Gautier 2015).

To describe the genetic drift of allele frequencies along the binary-branching paths, we modified the approach proposed in TreeMix (Pickrell and Pritchard 2012) and BayPass (Gautier 2015). In the Wright–Fisher model, the expected value and variance of allele frequency in *j*-th site are $E[\;f_{j}^{i}]=f_{A}^{i}$ and $var[\;f_{j}^{i}]\approx f_{A}^{i}(1-f_{A}^{i})t$, where *t* is a time variable. To match these first two moments after ilr transformation of allele frequencies (supplementary text S1, Supplementary Material online), the following should be satisfied: $E[x_{j}^{i}]=x_{A}^{i}$, $var[x_{j}^{i}]=\frac{t}{\;f_{A}^{i}(1-f_{A}^{i})}$.

Using the logic of model construction from TreeMix (Pickrell and Pritchard 2012) and Gaussian model for changing log-balances, we get that $x_{j}^{i}∼N\left(x_{A}^{i},\frac{t}{\;f_{A}^{i}(1-f_{A}^{i})}\right)$, where *t* is proportional to the cumulative path from the ancestral site to *j*-th site. Using Felsenstein's approach (Felsenstein 1973), we model the change of log-balances along the binary-branching path with multivariate normal distribution:


(1) $$\vec{x^{i}}∼MvN\left(\vec{x_{A}^{i}},\frac{s^{i}V}{f_{A}^{i}(1-f_{A}^{i})}\right),$$

where $\vec{x^{i}}=(x_{1}^{i},x_{2}^{i},\ldots x_{M}^{i})$, $s^{i}$ is the constant of proportionality specific for *i*-th SNP, and *V* is M×M matrix, which reflects the covariance structure between *M* sites. On the diagonal, matrix *V* contains cumulative branch lengths from the tree root to respective leaves, and the off-diagonal elements are equal to the sum of common branches for respective pair of sites (Felsenstein 1973). We compute values in *V* matrix based on the spread model and scale it, so that the mean value of diagonal elements should equal to one.

#### Prior Probabilities and Markov Chain Monte Carlo

For each SNP, model has the following parameters: the allele frequency in the ancestral population, log-balances of allele frequencies for *M* sites, and the constant of proportionality. To get estimates, we constructed Bayesian model with the following prior distributions for parameters.

For $f_{A}^{i}$, we proposed uninformative beta prior, $Beta(a^{i},b^{i})$, with uniform prior for the mean, $\frac{a^{i}}{a^{i}+b^{i}}∼Unif(0,1)$ and exponential prior for the so-called sample size, $a^{i}+b^{i}∼Exp(1)$. We also assume the exponential prior for constant of proportionality: $s^{i}∼Exp(1)$.

The complexity of the model does not allow the use of Gibbs sampling. Instead, we performed the algebraic inference of derivatives for log posterior distribution and run Hamiltonian Monte Carlo sampling algorithm (Neal 2012) in pyhmc (https://pythonhosted.org/pyhmc/) to get parameter estimates. For each chickpea population (specific region and market class), we ran 3 Markov chain Monte Carlo (MCMC) chains of length 50,000 and traced the Gelman–Rubin convergence diagnostic (<1.1) and effective sample size.

To conclude which model of chickpea dispersal within a region is more probable, we separately got estimates on *V* matrix calculated for estimated routes and linear distances. Then we compared BIC between two estimates (supplementary file S1, Supplementary Material online).

### Model for Migration between Clusters

The “migadmi” model describing migrations and adm**i**xtures of populations is implemented in Python package (https://github.com/iganna/migadmi). Testing of migadmi on simulated data is provided in the supplementary text S7, Supplementary Material online.

To test hypothetical migration routes of chickpea between regions, we created a model based on the same assumptions as used in the model for population spread within a region. We consider *P* populations characterized with vectors of log-balances of allele frequencies, which are obtained from the previous analysis. We denote log-balances of allele frequencies of *i*-th SNP in *j*-th populations with $x_{j}^{i}$.

A migration hypothesis is set by the binary tree, in which branch lengths are parameters. Based on the migration hypothesis, we construct the parametrized covariance matrix *V* and matrix *D* containing variances of differences between log-balances: $D_{\;jk\;}=V_{\;jj}+V_{kk}-2V_{\;jk}$. Then, we can construct the following likelihood function (supplementary text S2, Supplementary Material online):


(2) $$L(X|D)=\prod_{i=1}^{N}\prod_{\;j=1}^{P-1}\prod_{k=j+1}^{P}\;p_{N}(x_{j}^{i}-x_{k}^{i}|0,c^{i}D_{\;jk}),$$

where *N* is a number of SNPs, *X* is the matrix of log-balances for all SNPs and all populations, and $c^{i}$ is a SNP-specific scale parameter.

Likelihood (2) contains a unique scale parameter, $c^{i}$, for each SNPs, making the model overparametrized. To reduce the number of parameters, we applied the sliding window technique. We divided each chromosome into overlapping windows of the same size almost equal to the linkage disequilibrium, $3\cdot 10^{6}$ bp; the step parameter in the sliding window was $1\cdot 10^{6}$. As the density of SNPs along chromosomes is not uniform (supplementary fig. S6, Supplementary Material online), windows contained different numbers of SNPs; those with less than 10 SNPs were filtered out.

We assumed that SNPs within each window are probably linked and had evolved with a similar rate. This assumption allows us to avoid $c^{i}$ parameters (set it to 1) and infer objective function proportional to log-likelihood (see supplementary text S3, Supplementary Material online):


(3) $$f(D,w)\propto \sum_{\;j=1}^{P-1}\sum_{k=j+1}^{P}\log\;p_{N}(d_{w}(x,j,k)|0,D_{\;jk}),$$

where $d_{w}(x,j,k)$ is a root mean square distance between *j*-th and *k*-th populations, computed on SNPs from *w*-th window (see supplementary text S3, Supplementary Material online), and $\log\;p_{N}$ denotes the log-density of normal distribution. We estimate parameters in *D* matrix separately for each window.

### Modeling Admixture Events

We developed a new model of admixtures which considers that 1) admixture events happened long ago and all populations (both source and mixed) accumulated their own variance after the event; 2) the number of source populations in one event is not constrained, that is, can be higher than 2; 3) several admixture events can be analyzed simultaneously; and 4) admixtures can form a hierarchy, that is, a mixed population in one admixture event can be a source in another event.

Let population *y* be a mixture of *Q* sources ($z_{q},\;q=\bar{1,Q}$), which are precursors of *Q* current populations ($x_{q},\;q=\bar{1,Q}$). We parametrized this admixture event with the following variables: $t_{y}$, own variance of the mixed population; $w_{q}$, weights of source populations, $\sum_{q=1}^{Q}\;w_{q}=1$; and α∈[0,1], part of own variance of $x_{q}$ which is common with $z_{q}$ (see supplementary text S4, Supplementary Material online). To avoid overparameterization, we set the regularization on $w_{q}$ with the Dirichlet prior (all concentration parameters, *λ*, equal to 0.9).

To test an admixture hypothesis, we 1) constructed the corresponding tree with admixture events, 2) parametrized *V* and *D* matrices based on the tree, and 3) estimated parameters maximizing the objective function (4).


(4) $$\begin{array}{rl} f(D,w)\;\propto & \sum_{\;j=1}^{P-1}\sum_{k=j+1}^{P}\log\;p_{N}(d_{w}(x,j,k)|0,D_{\;jk})\\ & +(\lambda -1)\sum_{q=1}^{Q}\log\;w_{q}, \end{array}$$

## Supplementary Material

msad110_Supplementary_Data

## Data Availability

All Illumina data are available from the National Center for Biotechnology database under BioProject PRJNA388691. Processed initial data for the analysis are uploaded to GitHub repositories with the code.

## Code availability

Codes for the popdisp and migadmi analysis frameworks are available at https://github.com/iganna/popdisp and https://github.com/iganna/migadmi.
