# Supplementary material for: Historical Routes for Diversification of Domesticated Chickpea Inferred from Landrace Genomics
Source: Mol Biol Evol. 2023 May 9;40(6):msad110. doi: 10.1093/molbev/msad110 (PMC10285117; doi:10.1093/molbev/msad110)

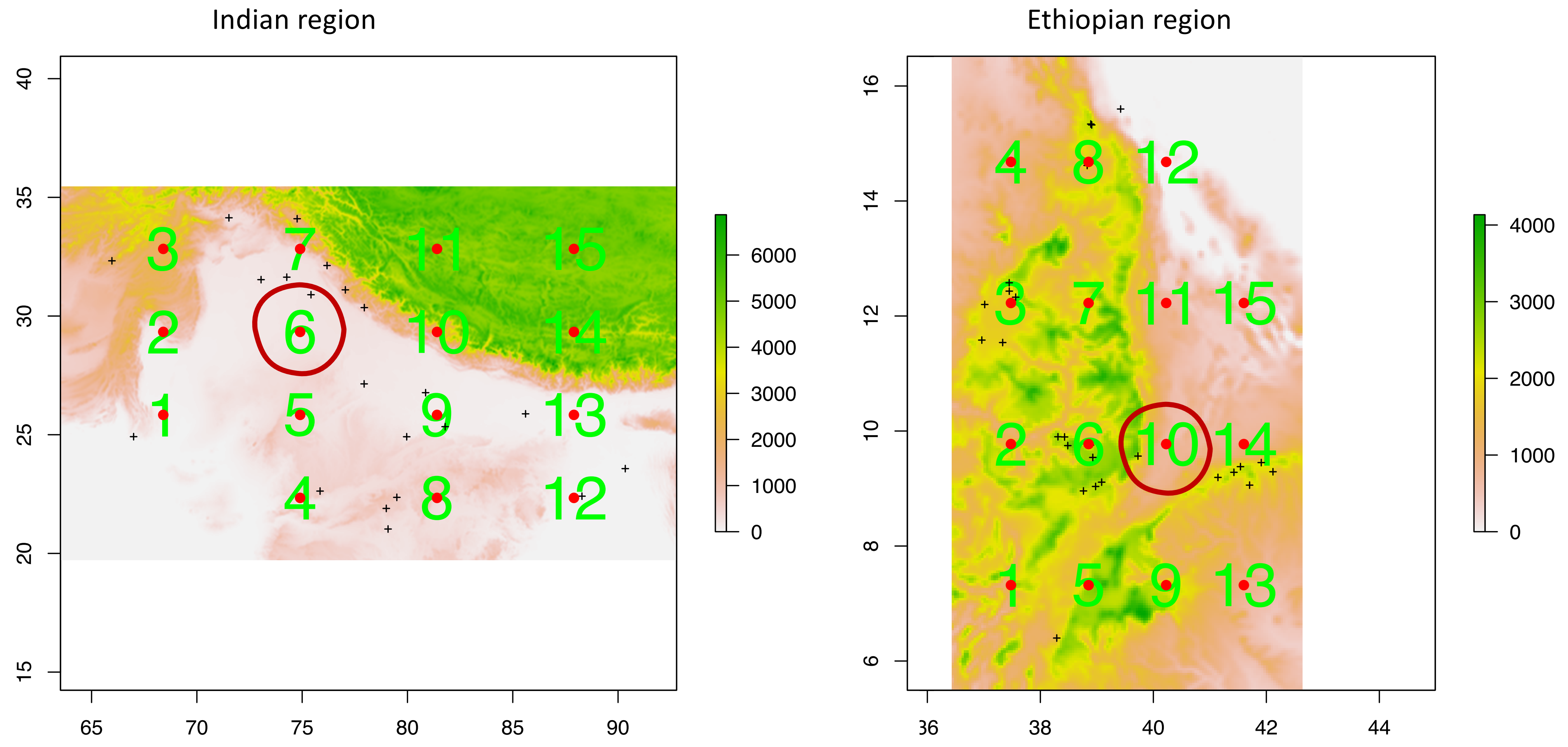

Indian region
Ethiopian region
40
35
30
25
20
15
65
70
75
80
85
90
6000
5000
4000
3000
2000
1000
0
16
14
12
10
8
6
36
38
40
42
44
4000
3000
2000
1000
0
1
2
3
4
5
6
7
8
9
10
11
12
13
14
15

Uzbek-west region

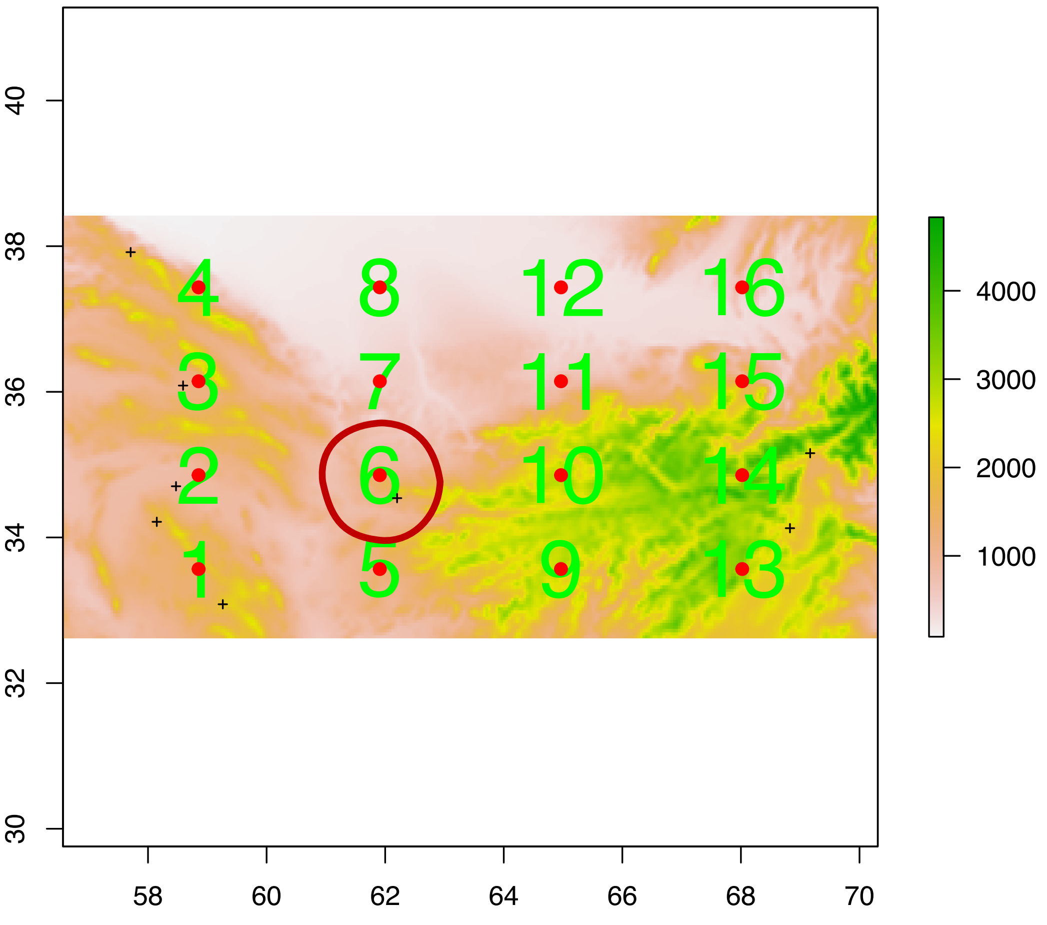

Uzbek-east region

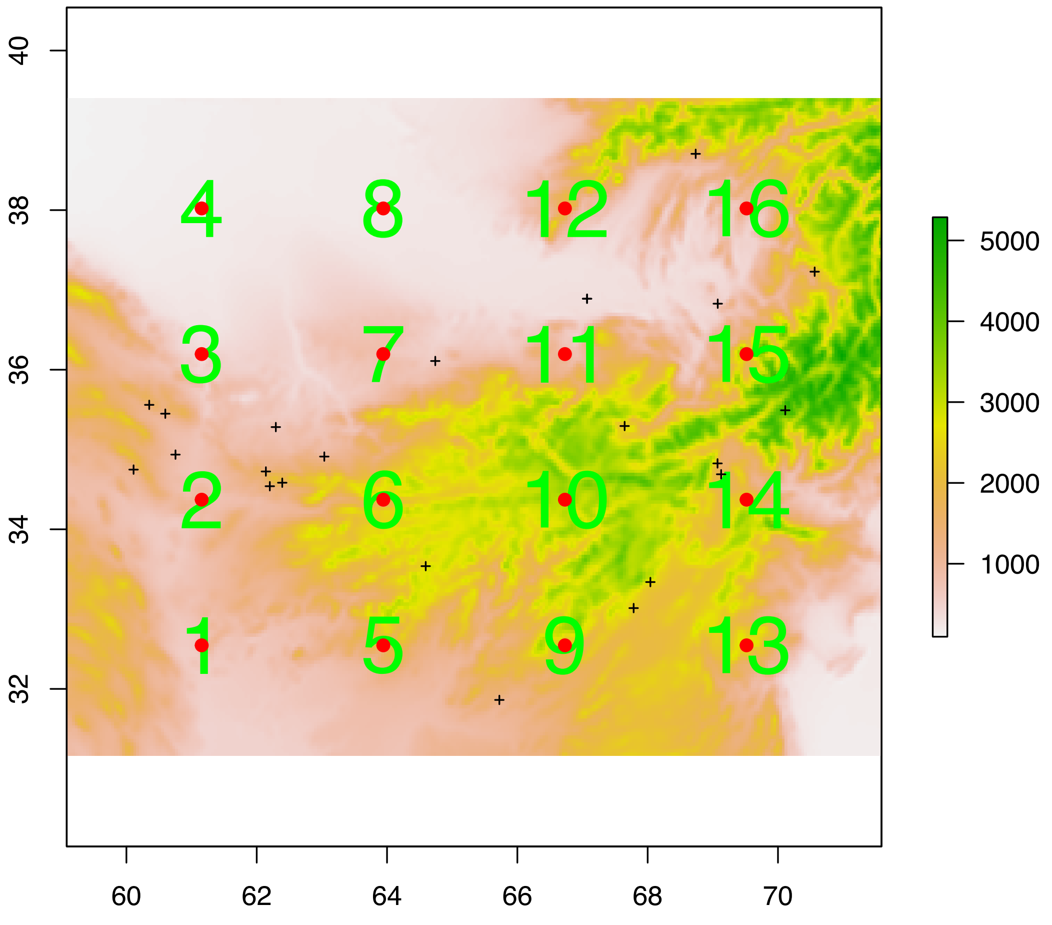

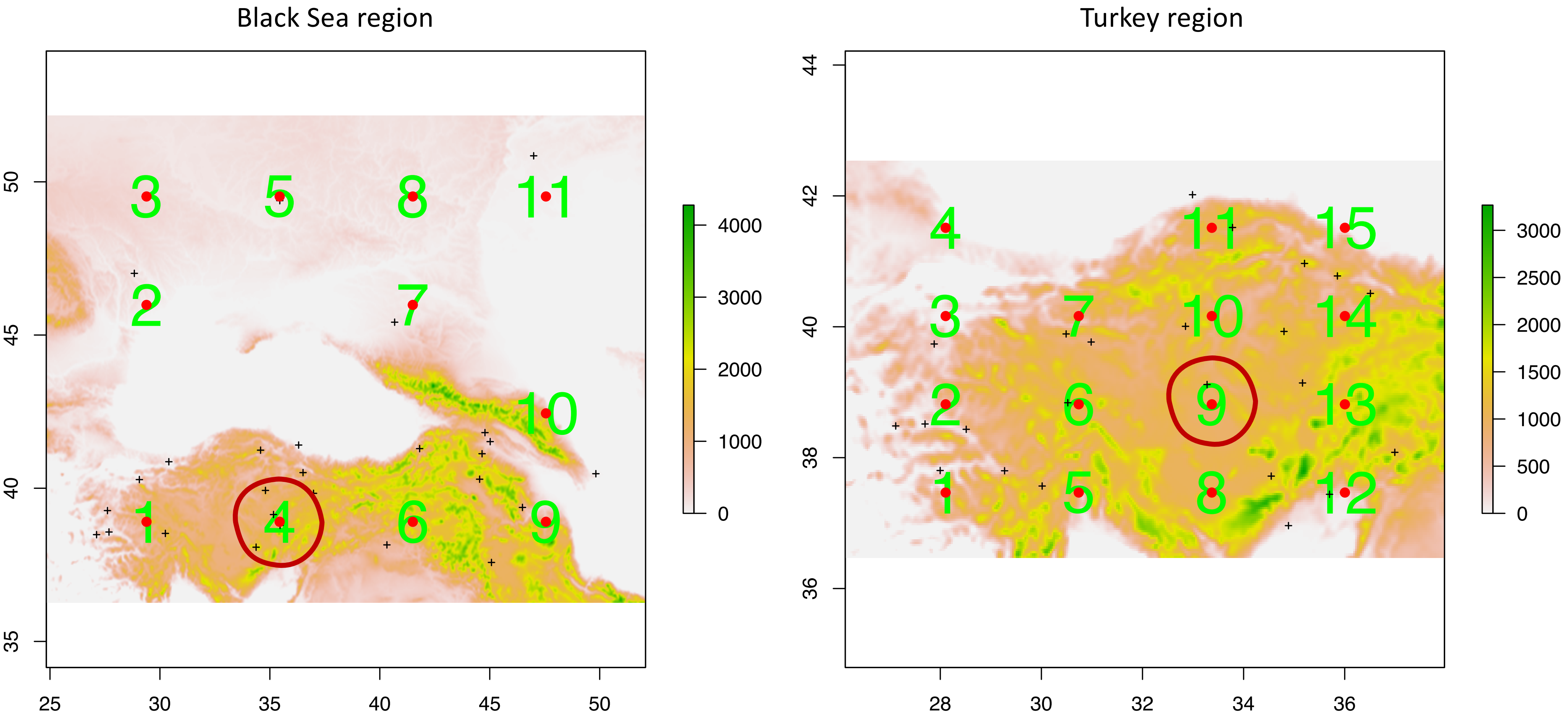

Black Sea region
Turkey region

Lebanese region

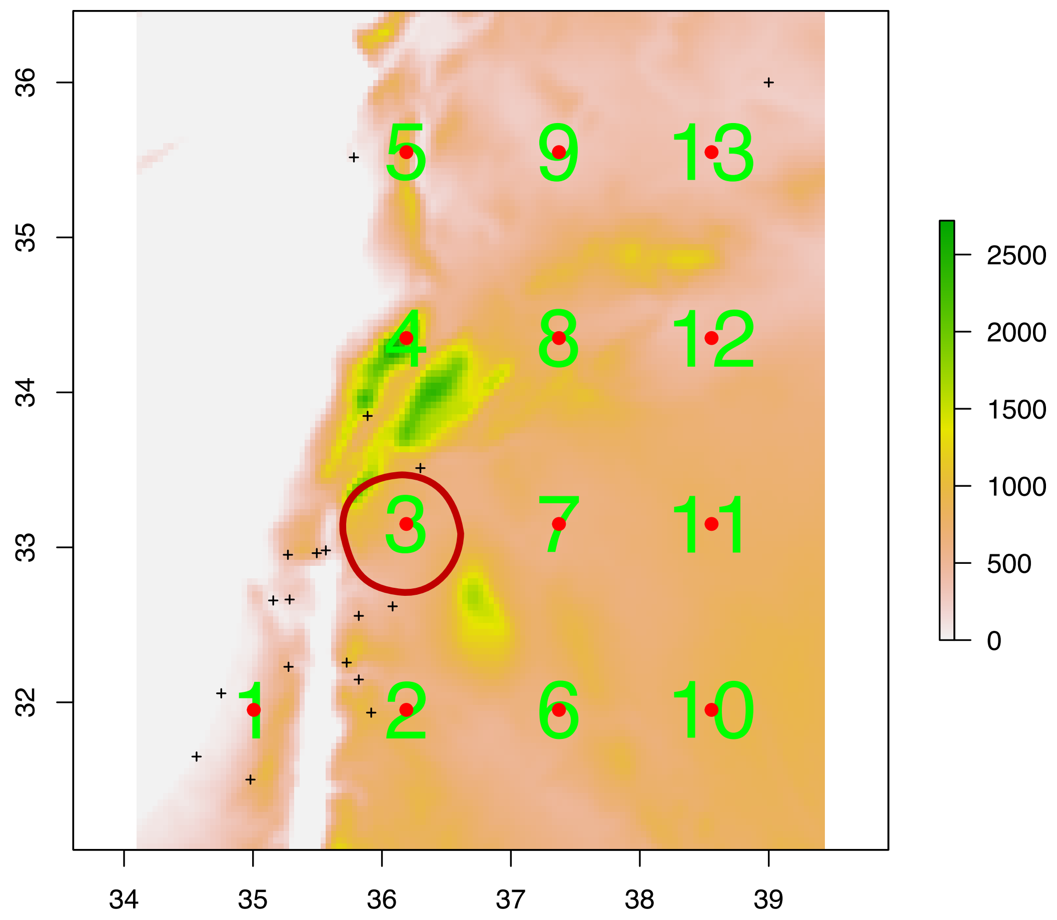

Supplement: msad110_Supplementary_Data [file msad110_supplementary_data.zip › Supplementary_File2.pdf]
