# Supplementary material for: Historical Routes for Diversification of Domesticated Chickpea Inferred from Landrace Genomics
Source: Mol Biol Evol. 2023 May 9;40(6):msad110. doi: 10.1093/molbev/msad110 (PMC10285117; doi:10.1093/molbev/msad110)
Supplement: msad110_Supplementary_Data [file msad110_supplementary_data.zip › Supplementary_text_fixed.pdf]

Supplementary text for "Historical routes for diversification of domesticated chickpea inferred from landrace genomics"

Anna A. Igolkina, Nina V. Noujdina, Margarita Vishnyakova, Travis Longcore,
Eric von Wettberg, Sergey V. Nuzhdin, Maria G. Samsonova

Supplementary text 1. First two moments of ilr-transformed allele frequencies.

Let a population be described by the frequency of alternative allele of a biallelic SNP, f. The population comes out from the ancestral one with the allele frequency f_A under the Wright Fisher model of genetic drift. In the Wright Fisher model, expected value and variance of allele frequency are $E[f] = f_A$, $var[f] = f_A(1 - f_A)\left(1 - \left(1 - \frac{1}{2N}\right)^{\tau}\right)$, where τ is the number of generations separating current and ancestral populations, and N is the size of diploid population. Using the Binomial approximation, $var[f] \approx f_A(1 - f_A)\frac{\tau}{2N} = f_A(1 - f_A)t$, where t can be considered as the amount of genetic drift.

We applied the ilr-transformation for allele frequencies and obtained $x = \log\frac{1-f}{f}$, $x_A = \log\frac{1-f_A}{f_A}$. These new variables mean the log-balance between reference and alternative allele frequencies in the current and ancestral populations. Using Taylor expansions, the second order approximation of the expected value of x is x_A, and the approximation of variance is the following:

$$var[x] = \left(\frac{d}{df_A}\left(\log\frac{1-f_A}{f_A}\right)\right)^2 \cdot var[f] = \left(\frac{1}{1-f_A} - \frac{1}{f_A}\right)^2 f_A(1 - f_A)t = \frac{t}{f_A(1-f_A)}.$$

Supplementary text 2. Estimates for branch parameters of a tree

Let's consider P populations originated from one ancestral state and a binary tree depicting their migration history; all tree branch lengths are parameters. Each population is characterized by log-balance of allele frequencies for a SNP, x_i. In the model for population spread within a region, it has been assumed that $\vec{x} \sim MvN\left(\overrightarrow{x_A}, \frac{sV}{f_A(1-f_A)}\right)$, where $\vec{x} = (x_1, x_2, \dots, x_P)$, x_A is the log-balance of allele frequency in the root of the tree (ancestral state). However, in testing historical hypotheses, there is no given information about the ancestral state: f_A is not known, position of the root in the binary tree is parametrized. Therefore, it is impractical to include f_A into the model and use the above-mentioned multivariate normal distribution.

To avoid the use of f_A, we propose an approach which considers total variance between populations instead of covariance. Let covariance matrix between populations, V be obtained based on the fully parametrized binary tree according to Felsenstein's method (Felsenstein, 1973) (see Example on Supplementary text 2 - Figure 1). Then, we can obtain a matrix D, which elements are proportional to variances of the difference between log-balances:

$$var(x_i - x_j) \propto D_{ij} = V_{ii} + V_{jj} - 2V_{ij}.$$

Based on Gaussian changing log-balances, we get:$(x_i - x_j) \sim \mathcal{N}(0, c \cdot D_{ij})$, where c is a constant of proportionality covering $\frac{s}{f_A(1-f_A)}$.

To get maximum likelihood estimates of the tree branch length based one SNP, the following likelihood function can be written:

$$\mathcal{L} = \prod_{i=1}^{P-1} \prod_{j=i+1}^{P} p_{\mathcal{N}}(x_i - x_j | 0, cD_{ij}).$$

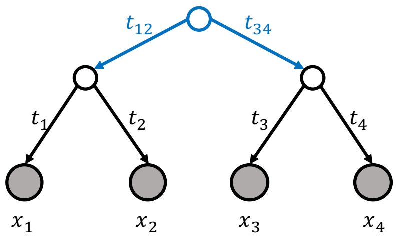

**V**	x_1	x_2	x_3	x_4
x_1	$t_1 + t_{12}$	t_{12}	0	0
x_2	t_{12}	$t_2 + t_{12}$	0	0
x_3	0	0	$t_3 + t_{34}$	t_{34}
x_4	0	0	t_{34}	$t_4 + t_{34}$

Supplementary text 2 - Figure 1. Example evolutionary tree for four populations; branches are parametrized. The table represents matrix V.

Supplementary text 3. Inference of likelihood function for a set of linked SNPs

A "window" is a segment on a chromosome of length equal to a predefined value (≈LD) that contains a subset of SNPs. We assumed, that, within each window, SNPs are probably linked and they had evolved with a similar rate. Let G_w be a set (group) of SNPs corresponding to w-th window, and s^w be a scale, specific for this window and reflecting the rate. For i-th SNP in j-th population, we denote log-balances of allele frequency with x_j^i. Then, the Likelihood function for log-balances of allele frequencies in the w-th window is:

$$\mathcal{L}(X|D,w) = \log\left(\prod_{i\in G_w}\prod_{j=1}^{P-1}\prod_{k=j+1}^{P} p_{\mathcal{N}}\left(x_j^i - x_k^i \middle| 0, \frac{s^w D_{jk}}{f_A^i(1-f_A^i)}\right)\right).$$

where f_A^i is the allele frequency of the ancestral state. This value is not a parameter, is not known, and plays the scale role. In line with CoDA, we estimate it as $\widehat{f_A^i} = 1/(1 + \exp(\operatorname*{mean}_j x_j^i))$. Let denote constant $q_i^2 = \widehat{f_A^i}(1-\widehat{f_A^i})$, then the likelihood is proportional to:

$$\mathcal{L}(X|D,w) \propto \prod_{i\in G_w}\prod_{j=1}^{P-1}\prod_{k=j+1}^{P} \frac{1}{\sqrt{2\pi s^w D_{jk}}}\exp\left(-\frac{\left(x_j^i - x_k^i\right)^2}{s^w D_{jk}/(q_i{}^2)}\right) =$$

$$\prod_{i\in G_w}\prod_{j=1}^{P-1}\prod_{k=j+1}^{P} \frac{1}{\sqrt{2\pi s^w D_{jk}}}\exp\left(-\frac{\left((x_j^i - x_k^i)q_i\right)^2}{s^w D_{jk}}\right) =$$

$$\prod_{j=1}^{P-1}\prod_{k=j+1}^{P} \frac{1}{\left(2\pi s^w D_{jk}\right)^{\frac{|G_w|}{2}}}\exp\left(-\frac{\sum_{i\in G_w}\left((x_j^i - x_k^i)q_i\right)^2}{s^w D_{jk}}\right) =$$

$$\prod_{j=1}^{P-1}\prod_{k=j+1}^{P}\left[\frac{1}{\left(2\pi s^w D_{jk}\right)^{\frac{1}{2}}}\exp\left(-\frac{\frac{1}{|G_w|}\sum_{i\in G_w}\left((x_j^i - x_k^i)q_i\right)^2}{s^w D_{jk}}\right)\right]^{|G_w|} =$$

$$\left[\prod_{j=1}^{P-1}\prod_{k=j+1}^{P} p_{\mathcal{N}}\left(d_w(x,j,k)\middle|0, s^w D_{jk}\right)\right]^{|G_w|},$$

where $d_w(x,j,k) = \sqrt{\frac{\sum_{i\in G_w}\left((x_j^i - x_k^i)q_i\right)^2}{|G_w|}}$ is the normalized root mean square distance between j-th and k-th populations, computed on SNPs from w-th window. However, as matrix D is fully parametrized, we can set $s^w = 1$ without loss of generality. To get parameters estimated, we can remove the power and maximize the following log-likelihood function:

$$\log \mathcal{L}(X|D,w) \propto \sum_{j=1}^{P-1} \sum_{k=j+1}^{P} \log p_{\mathcal{N}}\big(d_w(x,j,k)\big|0, D_{jk}\big).$$

Supplementary text 4. Identification of parameters in the mixture model

Consider six populations originated from one ancestral state, and a tree depicting the history of the populations (Supplementary text 4 - Figure 1a); x_j is a normal random variable reflecting the log-balance of frequencies for the SNP in population j (Supplementary text 4 - Figure 1a). We denote lengths of tree branches with t_i.

Let the seventh population (having y log-balance of frequencies for the SNP) originate by a mixture event of three populations (precursors of x_1, x_3, and x_6), and then evolve independently along the branch with the length t_y (Supplementary text 4 - Figure 1b). We assume that the mixture event happened long ago, so that current populations x_i have their own evolutionary history, independent from the sources z_i. To carefully consider the mixture event, we introduced weight parameters w_i, α_i, β_i, as demonstrated in Supplementary text 4 - Figure 1b,e. In our example, the number of additional parameters is 10, and the number of constraints is 4; hence, the number of free parameters is 6. The number of cells in the matrix D, which contain additional parameters, is 6, so all free parameters are identifiable in this example. However, in the extreme situation, when all six initial populations can be considered as sources of the mixed one, the number of free parameters reaches 12, and some of them become non-identifiable.

In general, when the initial tree connects n_{pop} populations and all of them can be sources of a mixed one, the number of free parameters is $2n_{pop}$ and number of cells in the matrix D, which contain additional parameters, is n_{pop}. Therefore, to avoid this overparameterization we introduce several constraints. First, we assume that all α_i are equal, and this assumption reduce the number of free parameters to $(n_{pop} + 1)$ (Supplementary text 4 - Figure 1c). Second, we set the regularization on w_i weights using the Dirichlet prior with all concentration parameters equal to 0.9: $\left(w_1, \dots w_{n_{pop}}\right) \sim \text{Dirichlet}(0.9 \dots 0.9)$. Imitating absorbing states in the genetic drift, this prior tends to pull some weights to zeros, i.e. to put $\left(w_1, \dots w_{n_{pop}}\right)$ vector closer to the border of n_{pop}-dimensional simplex. These two introduced restrictions make all free parameters in the model identifiable.

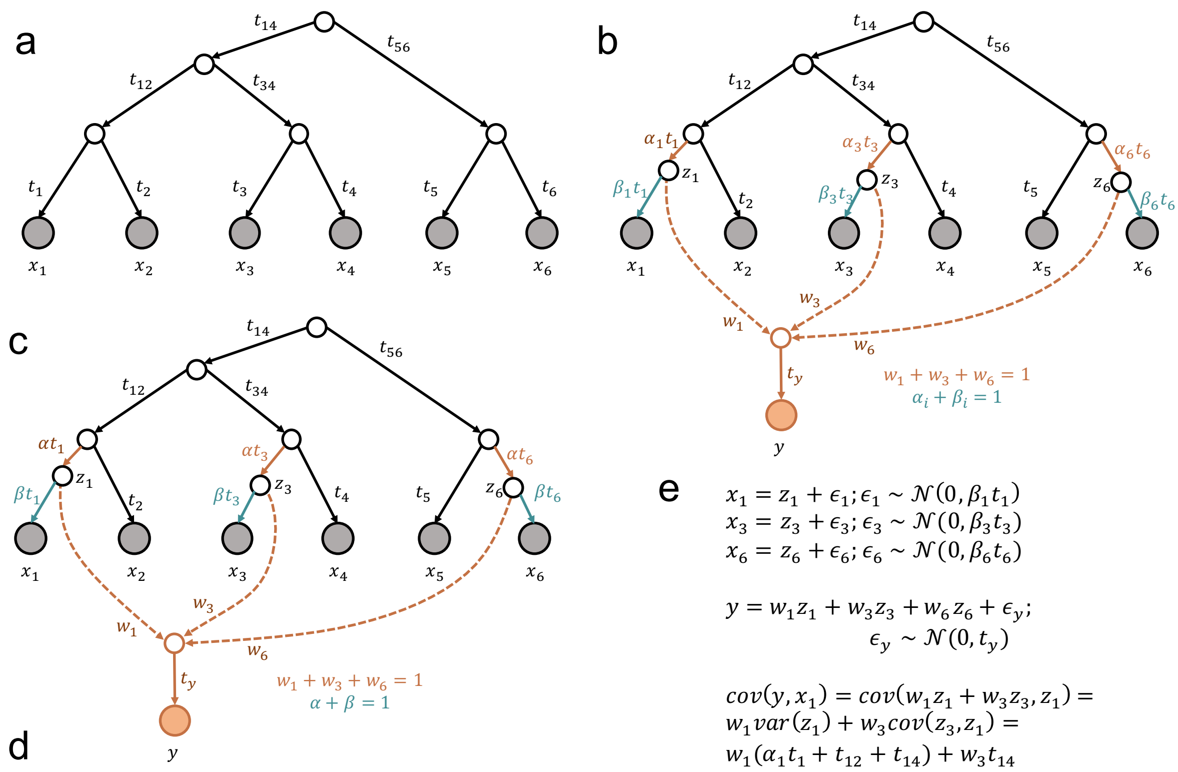

e

$$x_1 = z_1 + \epsilon_1; \epsilon_1 \sim \mathcal{N}(0, \beta_1 t_1)$$
$$x_3 = z_3 + \epsilon_3; \epsilon_3 \sim \mathcal{N}(0, \beta_3 t_3)$$
$$x_6 = z_6 + \epsilon_6; \epsilon_6 \sim \mathcal{N}(0, \beta_6 t_6)$$

$$y = w_1 z_1 + w_3 z_3 + w_6 z_6 + \epsilon_y; \quad \epsilon_y \sim \mathcal{N}(0, t_y)$$

$$cov(y, x_1) = cov(w_1 z_1 + w_3 z_3, z_1) = w_1 var(z_1) + w_3 cov(z_3, z_1) = w_1(\alpha_1 t_1 + t_{12} + t_{14}) + w_3 t_{14}$$

d

V	x_1	x_2	x_3	x_4	x_5	x_6	y
x_1	$t_1 + t_{12} + t_{14}$	$t_{12} + t_{14}$	t_{14}	t_{14}	0	0	$\alpha w_1 t_1 + w_1 t_{12} + (w_1 + w_3) t_{14}$
x_2	$t_{12} + t_{14}$	$t_2 + t_{12} + t_{14}$	t_{14}	t_{14}	0	0	$w_1 t_{12} + (w_1 + w_3) t_{14}$
x_3	t_{14}	t_{14}	$t_3 + t_{34} + t_{14}$	$t_{34} + t_{14}$	0	0	$\alpha w_3 t_3 + w_3 t_{34} + (w_1 + w_3) t_{14}$
x_4	t_{14}	t_{14}	$t_{34} + t_{14}$	$t_4 + t_{34} + t_{14}$	0	0	$w_3 t_{34} + (w_1 + w_3) t_{14}$
x_5	0	0	0	0	$t_5 + t_{56}$	t_{56}	$w_6 t_{56}$
x_6	0	0	0	0	t_{56}	$t_6 + t_{56}$	$\alpha w_6 t_6 + w_6 t_{56}$
y	$\alpha w_1 t_1 + w_1 t_{12} + (w_1 + w_3) t_{14}$	$w_1 t_{12} + (w_1 + w_3) t_{14}$	$\alpha w_3 t_3 + w_3 t_{34} + (w_1 + w_3) t_{14}$	$w_3 t_{34} + (w_1 + w_3) t_{14}$	$w_6 t_{56}$	$\alpha w_6 t_6 + w_6 t_{56}$	$\alpha^2 w_1^2 t_1 + \alpha^2 w_3^2 t_3 + \alpha^2 w_6^2 t_6 + w_1^2 t_{12} + w_3^2 t_{34} + (w_1 + w_3)^2 t_{14} + w_6^2 t_{56} + t_y$

Supplementary text 4 - Figure 1. An example tree describing the evolutionary history of 7 populations with one admixture. (a) Base tree of 6 non-admixed populations. (b) Admixture event with parametrizations. (c) Reduction of parameters in the admixture event. (d) The table represents matrix V. x_i represent the frequency balance of a SNP for i-th population, y is the population formed with an admixture, t_i are the length of a tree branch, w_i, α_i, and β_i are weight parameters. The V-table demonstrates the variance-covariance matrix V for all populations after re-parametrization.

Supplementary text 5. Comparison of migadmi results with TreeMix, MixMapper and qpGraph

Supplementary text 5-Table 1. Comparison of admixture methods

	migadmi	TreeMix	MixMapper	qpGraph/fing_graph in admixtools
Search for admixtures of >2 sources	+	-	-	-
Several non-nested admixtures	+	+	-	+
Number of nested admixtures	≥1	0	2	>1
Adding admixture event to a core tree	+	-	+	-
Admixture pattern along the chromosome	+	-	-	-
Can take the tree as input	+	+	-	(as initial point for optimization)
Accounting for own evolutionary history for both mixed population and source populations	+	-	-	+
Compositional data analysis	yes	no	no	no

To estimate the migration and admixture events in our study, we developed a new method, **migadmi**, because of the limitations of the existing ones, TreeMix (Pickrell and Pritchard, 2012) and MixMapper (Lipson et al., 2013). We created a list of characteristics to compare the packages and found that our method covers and outperforms capabilities of TreeMix and MixMapper: our package copes with estimating multiple complex admixture events with more than 2 sources and demonstrates the admixture patterns along the chromosomes. Moreover, it has two additional features that were not accounted for in previous models.

The first feature is that **migadmi** allows populations to get their own variance after admixture events. In the existing approaches, it is assumed that the composite population is a weighted

sum of some source populations, and weights sum to 1. However, in reality, almost no population is settled as a net sum of two or more. Ordinarily, when a part of one population appears in a new place, it evolves some period of time getting its own variability, and then if the admixture event happens, the mixed population continues to evolve. As a result, the variance in the admixed population can be factored into contributions from source populations and self-accumulated variance. The latter is especially important if the admixture events happened long ago (e.g., as in our study). Things get more complicated when considering that source populations have also evolved. To avoid modeling the mixed populations as a weighted sum of source ones, we parametrized the own variance of each population after the admixture event.

The second important feature of **migadmi** is the use of ilr-transformed allele frequency instead of allele frequency itself. Allele frequencies, as fractions or percentages, are constrained (i.e. sum up to 1 or 100%), which makes standard statistical methods inapplicable. For example, frequencies cannot be modelled as normally distributed random variables, as the domain of the normal distribution is $(-\infty, +\infty)$, not $[0, 1]$. Another problem is presence of negative bias in covariance estimates between frequencies (Aitchison, 1986). Moreover, frequency of one allele is inextricably linked with frequencies of others as they sum to 1. Therefore, modeling frequency changes of one allele cannot be considered without modeling changes in other alleles. To correctly work with frequencies, the theory of compositional data analysis and Aitchison geometry were first established in the end of previous century (Aitchison, 1986; Pawlowsky-Glahn and Buccianti, 2011) Following this theory, one can independently analyze $(D-1)$ balances between frequencies, instead of D frequencies. In case of biallelic SNPs, the balance is the logarithm of the ratio between reference and alternative alleles, and this balance takes values in $(-\infty, +\infty)$. We adapted the use of balances to model changes of allele frequencies in line with the Wright-Fisher drift model. The balance-based approach was used in both **popdisp** and **migadmi** models.

The direct comparison of migadmi results with TreeMix and MixMapper results is not possible because we used migadmi to estimate complex admixture graphs, which TreeMix and MixMapper cannot cope with (Supplementary text 5-Table 1). However, we performed the standard TreeMix and MixMapper analyses and traced the common and different trends in results.

First, we applied TreeMix and set to estimate 4 events within 10 populations. We used TreeMix without tree root specification (Supplementary text 5 - Figure 1). We also used the bootstrap with the size of 35, that equals to the mean number of SNPs in our sliding window technique. Obtained admixture graph demonstrated two expectable distant clades in trees: Uzbekistan-India and Turkey-Lebanon-BlackSea. (Supplementary text 5 - Figure 1a). TreeMix graphs partly support the hypothetical origin of Ethiopian and Uzbek-west desis. The location of Ethiopian

desi on the tree demonstrated its sources from both main clades, which is in line with the mixed origin of this population. The Uzbek-west desi population is connected between Turkish and Indian populations.

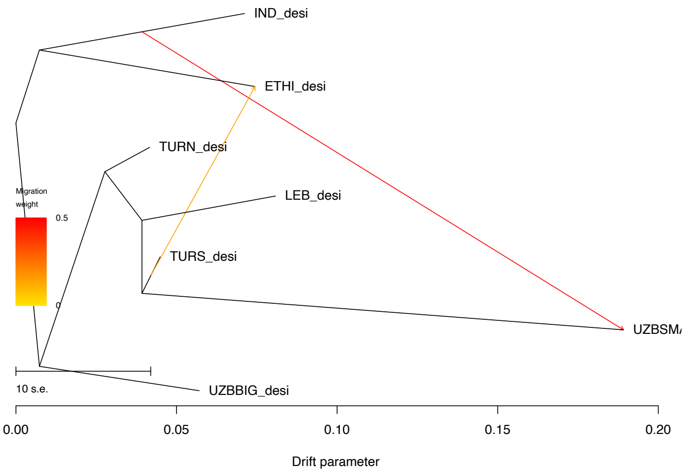

Supplementary text 5 - Figure 1. Admixture graphs obtained with the TreeMix package. TreeMix first estimates the tree based on all input populations (black branches), and then it introduces admixture events (colored arrows). Color of lines reflects the weight of the admixture from 0 to 0.5. TUSN means Black sea population, TURS – Turkey. UZBBIG – Uzbek-east, UZBSMALL – Uzbek-west, ETHI – Ethiopian, IND – Indian.

MixMapper takes source populations as input, then creates a tree of them and tests a mixed population adding it to the tree. We applied MixMapper in the bootstrap mode to match windows from our analysis. We analyzed the origin of Ethiopian desi, taking Turkish, Lebanese, Black Sea, Indian, Uzbeki-east desis as source populations. MixMapper revealed two sources of Ethiopian desi: Black Sea desi (74%) and Indian desi (26%). The direct analysis of Uzbek-west desi as a mixture from Turkish, Lebanese, Blask sea, Indian, Uzbeki-east desis revealed that it is as a mixture from Lebanese desi (68%) and Uzbek-east desi (32%).

To test the origin of kabuli, we tested two models and compared the admixture coefficients. In the first model, we assumed that Turkish, Lebanese, Black Sea and Indian, Uzbeki desis, and Turkish kabuli are six source populations, and Uzbeki, Lebanese and Black Sea kabuli are mixtures. The direct analysis revealed that Uzbeki kabuli has 69% from Uzbeki desi and 31% from [Turkish kabuli, Turkish desi, and Lebanese desi] clade. Using the same source populations, Lebanese kabulis were identified as 61% of Lebanese desis and 39% of Turkish kabulis; Black Sea kabulis – as 90% Black Sea desis and 10% of Turkey kabulis. In the second model, we assumed that Turkish, Lebanese, Black Sea, Indian, Uzbek-east desis, and Uzbek-east kabuli are six source populations, and Turkish, Lebanese and Black Sea kabulis are mixtures. In this case, we found that (i) Turkish and Lebanese kabulis are mixtures of Turkish

and Lebanese desis, (ii) Black Sea kabuli is a mixture of Turkish and Black Sea desis. Therefore, we may conclude that origin of kabuli is likely in the Turkey region.

To compare migadmi results with the admxitools, we ran two functions from the latter: qpgraph() and fing_graph(). The former function estimates the values of parameters for a given admixture graph, while the later one searches for the optimal admixture graph structure. To compare migadmi and admxitools graphs, we estimated scores of the graphs constructed by migadmi using qpgraph() and then ran find_graph() using as initial points either migadmi graphs or random seeds.

At first, we tried to construct the admixture graph for 5 desi populations with one admixture (Supplementary text 5 - Figure 2A). The score of the migadmi graph was the same as the score for the optimal topology from the find_graph() when the migadmi graph was provided as the initial point (Supplementary text 5 - Figure 2B). Two random runs of the find_graph() returned topologies with the less optimal scores (the lower score – the better) and questionable topologies: either the the Indian population or the Black Sea population were predicted as admixed (Supplementary text 5 - Figure 2C).

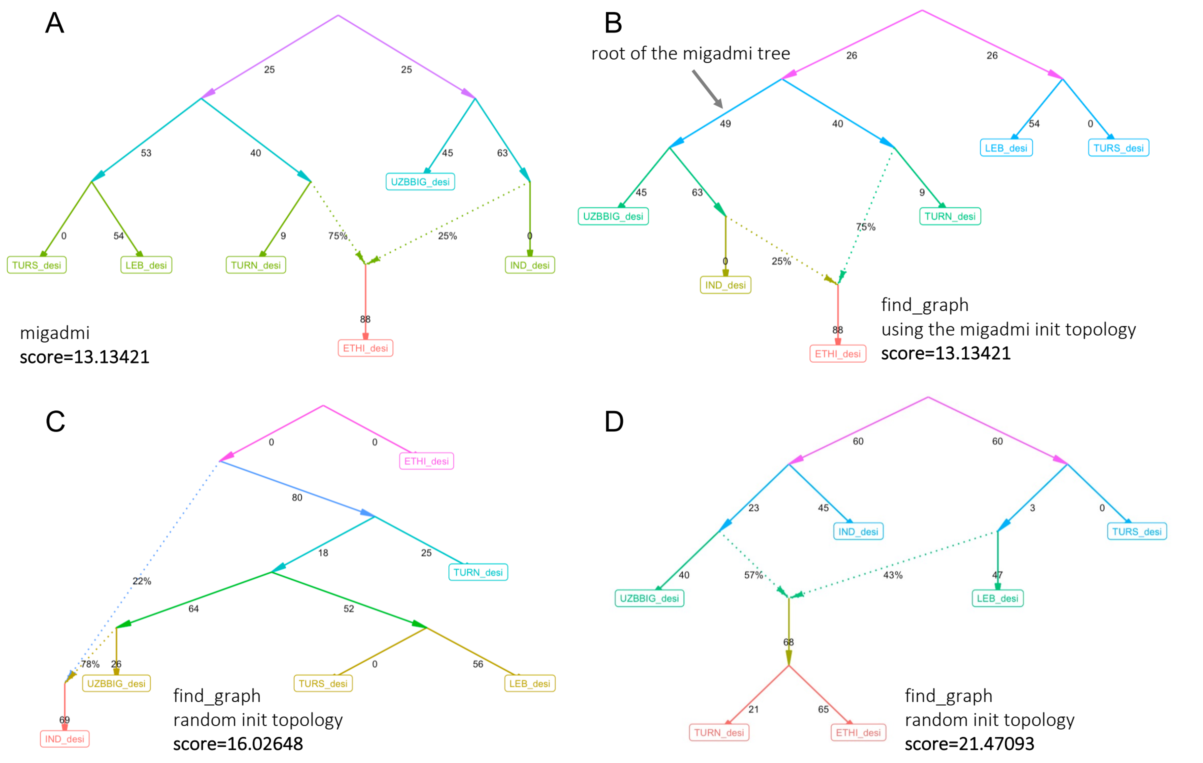

Supplementary text 5 - Figure 2. Topologies of the admixture graphs for six desi populations and one admixture. TURN means Black sea population TURS – Turkey population; UZBBIG – Uzb-east. (A) The admixture graph topology was suggested by migadmi. (B) Optimal admixture graph from the find_graph(), starting graph topology is on subfigure A. (C) Optimal admixture graph from the find_graph(), random seed = 30. (D) Optimal admixture graph from the find_graph(), random seed = 239.

Next, we repeated calculations with the Uzbek-west population as the second admixture. Based on find_graph() random runs, the migadmi graph topology was semi-optimal (Supplementary text 5 - Figure 3A,B). The most optimal solutions from the find_graph() suggested the Uzbek-west as the admixture of the Indian and Lebanese populations (Supplementary text 5 - Figure 3B,C), which is less probable than our finding, that Uzbek-west is the admixture of the Uzbek-east and Lebanese populations. Besides, some optimal topologies from the find_graph() were worse than the migadmi topology in terms of both score and logic (Supplementary text 5 - Figure 3D).

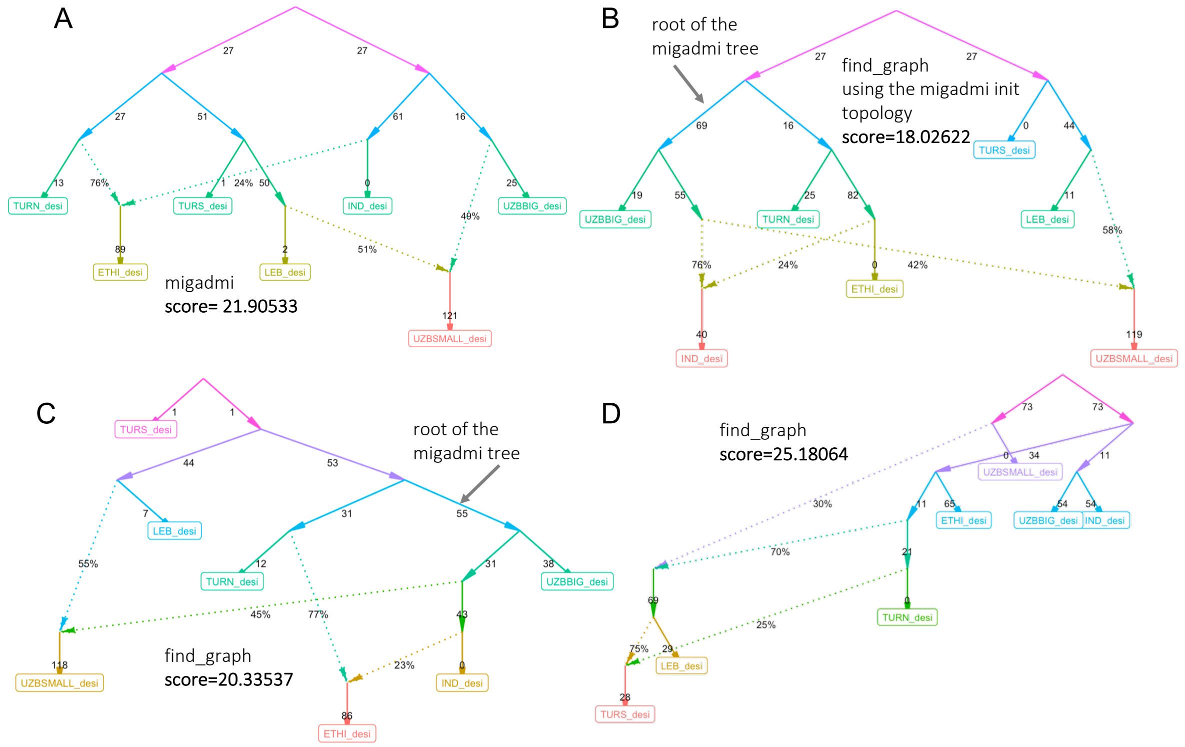

Supplementary text 5 - Figure 3. Topologies of the admixture graphs for seven desi populations and two admixtures. TURN means Black sea population TURS – Turkey population; UZBBIG – Uzb-east; UZBSMALL – Uzb-west. (A) The admixture graph topology was suggested by migadmi. (B) The optimal admixture graph from the find_graph(), starting graph topology is on subfigure A. (C) The optimal admixture graph from the find_graph(), random seed = 30. (D) The optimal admixture graph from the find_graph(), random seed = 239.

We were unable to directly compare topologies constructed for kabili populations using migadmi and find_graph(), as the comparison involves one population as a source for three admixtures, which looks like trifurcation on the graph. In such a situation find_graph() returns an error. However, we ran find_graph() several times for 5 desi and 4 kabuli populations to find

1, 2, or 3 admixtures, and relatively often, optimal graphs showed the Uzbek-east desi population as the admixed one in line with our findings (Supplementary text 5 - Figure 4).

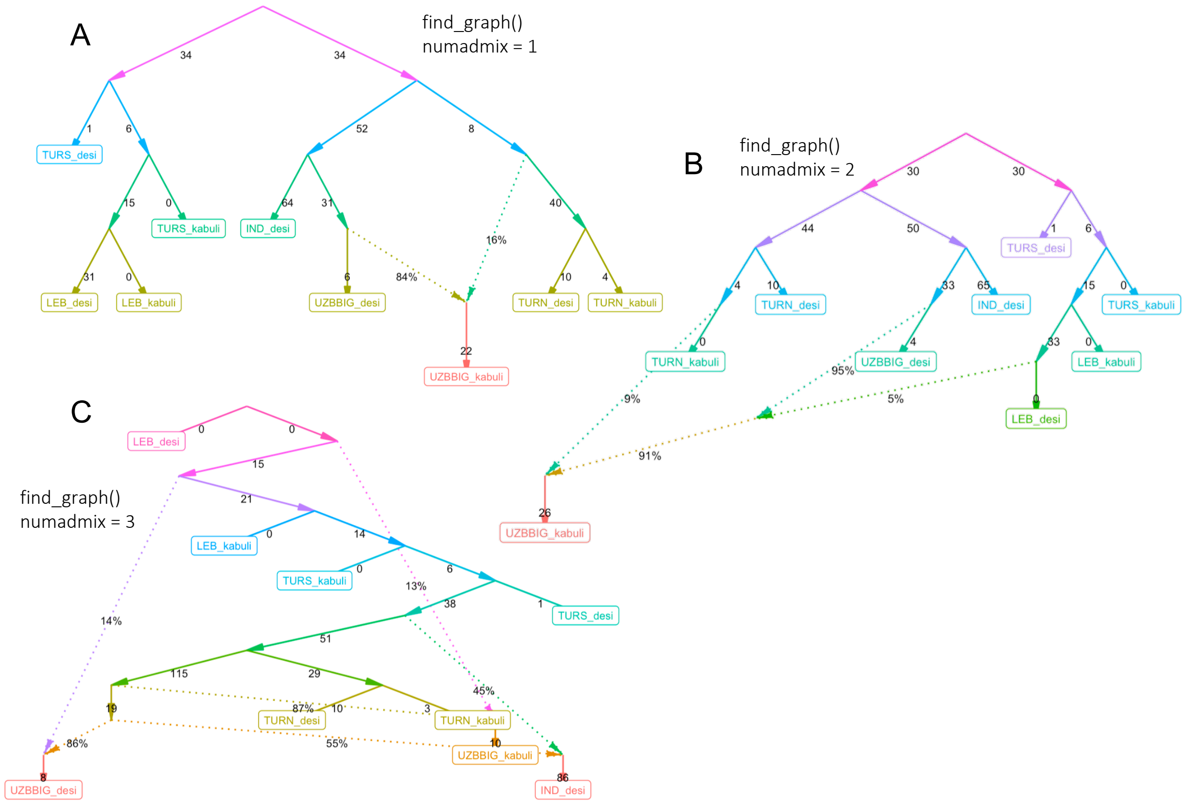

Supplementary text 5 - Figure 4. Topologies of the admixture graphs for seven desi populations and two admixtures. TURN means Black Sea population TURS – Turkey population; UZBBIG – Uzb-east. (A) Optimal admixture graph from the find_graph() assuming one admixture event. (B) Optimal admixture graph from the find_graph() assuming two admixture events. (C) Optimal admixture graph from the find_graph() assuming three admixture events.

Supplementary text 6. Artificial datasets for popdisp

Testing of the popdisp method consisted of three parts. First, we demonstrated that the popdisp prefers one of two types of sample dispersals correctly. Second, popdisp's estimates of frequencies are more accurate than those obtained with other estimation techniques. We created artificial datasets, that mimic real dispersals of samples in a geographic region from one center towards sampling sites. Third, we tested the sensitivity and robustness of the methods.

For testing, we developed a scheme to simulate datasets. The simulation has the following major parameters: the number of sites in the region, initial allele frequencies in the center, the range of possible numbers of samples per site. For simulation, we first generate a random rooted binary tree with the number of leaves equal to the number of sites. After that, we draw this tree on a 2D surface in the "equal daylight" layout (Supplementary text 6 - Figure 1a), split each branch into pieces, and slightly and randomly move each connection points between pieces (Supplementary text 6 - Figure 1b). Then, we randomly put into the tree's area five unfavorable zones with mimic mountains or lakes, i.e., areas, which are hard to move through (Supplementary text 6 - Figure 1c). Each zone was presented as a symmetric bivariate normal distribution around the zone center, and all zones together formed a landscape, where we allowed points between path's pieces to move on. The movement of a point was arranged by iterations in the gradient descend algorithm on the landscape. As a result, paths looked natural and emulated dispersals from the center towards sites on a geographic landscape (Supplementary text 6 - Figure 1c). When paths were created, we simulated 99 SNPs per path with initial allele frequencies varying from 0.01 to 0.99 with 0.01 step (Supplementary text 6 - Figure 1d). At last, we randomly generated the number of samples (alleles) within each sampling site and assigned 0/1 alleles to them according to the binomial distribution with simulated frequencies.

We simulated 100 paths of 30 sampling sites (the average number of sites per region in the chickpea dataset), created covariance matrices for them in "routes" and "linear" modes, and generated allele frequencies in sites. We created the first dataset to demonstrate that the popdisp correctly distinguishes between "routes" and "linear" distances and is more robust in estimates than the standard averaging. We generate numbers of samples in sites from the uniform distribution on [1, 4] and randomly assign one outlier site with the number of samples equal to 30. For all simulations, the Bayes factor for "routes" versus "linear" modes was higher than zero (Supplementary text 6 - Figure 1e), so that popdisp correctly identified routes as a more probable dispersal way. Then we calculated average allele frequencies across all samples in all sites, which is a standard way to get allele frequency estimates. These estimates were less accurate than those obtained by popdisp because the mean was sensitive to the outlier (Supplementary text 6 - Figure 1e). Estimates from the "linear" mode were also more accurate

than average estimates but less accurate than “routes” estimates (Supplementary text 6 - Figure 1e).

We also tested the sensitivity of the popdisp to the differences in sample sizes. For this purpose, we independently generated three datasets on previously simulated topologies, varying the number of samples in sites. The accuracy analysis demonstrated that even if each site is presented by one or two samples (alleles), the average difference between estimated and actual allele frequencies in the center is 0.05. When the number of samples varies between 1 and 20, the difference decreases to 0.03 (Supplementary text 6 - Figure 1g).

We also generate two additional tests for sensitivity and robustness. First, to evaluate the sensitivity of the popdisp to the number of sites, we randomly shortened the first dataset to 10 or 20 samples (1/3 or 2/3 of all samples). Second, to mimic possible noises of the data (for example, small unaccounted admixtures, which can be real in sites or artificial in the lab), we altered the first dataset shuffling generated alleles in 5 or 10 sites (1/6 and 1/3 of all sites). In all cases, the popdisp method predicts the allele frequencies in the region’s center with an error of less than 0.1 (Supplementary text 6 - Figure 1g). We suppose that this high robustness of the popdisp method is caused by connecting all samples into a network of paths (here, tree-like structure).

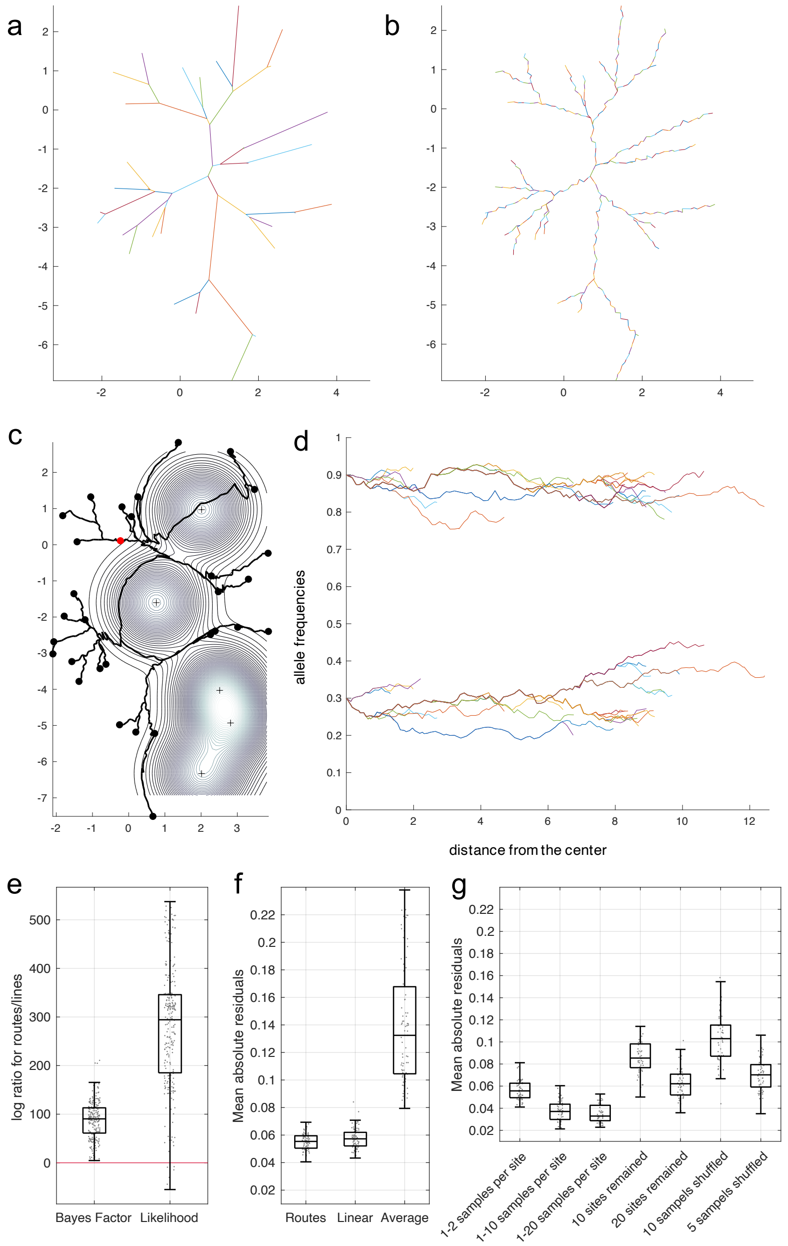

Supplementary text 6 - Figure 1. Simulation of a dataset for testing popdisp. (a) Drawing of a tree on a 2D surface in the "equaldaylight" layout. Colors are randomly assigned to branches. (b) Branches of the tree are split into pieces, colors are randomly assigned to pieces. (c) Landscape with five unfavorable zones. Crosses are centers of zones; contour lines join points of equal "height" in the landscape. Black dots are the final locations of sampling sites, the red dot is the final locations of the center of the region. Piecewise lines are routes from the center towards locations of sampling sites. (d) Two examples of the simulation of allele frequencies along the obtained dispersal structure (initial frequencies are 0.3 and 0.9). Colors represent paths from the center towards different sites. (e) Bayes factor (log-ratio) between "routes" and "linear" estimates. Log ratio of likelihood values between "routes" and "linear" estimates. (f) Mean absolute values of differences between estimated and actual frequencies in the centers. (g) Mean absolute values of differences between estimated and actual frequencies in the centers for data generated on different scenarios.

Supplementary text 7. Testing the migadmi

The input data for the migadmi is the distance matrix between sites. In our case, we estimate it on allele frequencies; however, users can calculate it on any other traits. We tested whether the migadmi accurately estimates values of parameters by given distance matrix and the admixture events. We generated simulated datasets as following. We started from 4 populations, united with a binary tree, simulated three nested admixture events of 2-3 sources, generated a distance matrix based on the simulation, used it as input for the migadmi. Then we optimized parameters and compared estimated and actual wights of admixtures. We generated 100 simulations, calculated the average absolute difference between weight parameters for each simulation, and the mean value of these differences was less than 0.001 (Supplementary text 7 - Figure 1). This result indicates that our is accurate and is limited by optimization method (SLSQP in our case) or possible structural unidentifiability of parameters.

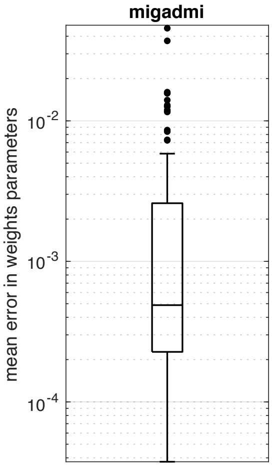

Supplementary text 7 - Figure 1. Mean differences between actual and estimated values of wight parameters of admixtures. Each point is one simulation.

References

Aitchison J. 1986. The Statistical Analysis of Compositional Data, London ; New York : Chapman and Hall.

Felsenstein J. 1973. Maximum-likelihood estimation of evolutionary trees from continuous characters. *Am J Hum Genet* **25**:471–492.

Lipson M, Loh P-R, Levin A, Reich D, Patterson N, Berger B. 2013. Efficient Moment-Based Inference of Admixture Parameters and Sources of Gene Flow. *Mol Biol Evol* **30**:1788–1802. doi:10.1093/molbev/mst099

Pawlowsky-Glahn V, Buccianti A. 2011. Compositional Data Analysis. Chichester, UK: John Wiley & Sons, Ltd. doi:10.1002/9781119976462

Pickrell JK, Pritchard JK. 2012. Inference of Population Splits and Mixtures from Genome-Wide Allele Frequency Data. *PLoS Genet* **8**:e1002967. doi:10.1371/journal.pgen.1002967
