# Supplementary material for: Historical Routes for Diversification of Domesticated Chickpea Inferred from Landrace Genomics
Source: Mol Biol Evol. 2023 May 9;40(6):msad110. doi: 10.1093/molbev/msad110 (PMC10285117; doi:10.1093/molbev/msad110)
Supplement: msad110_Supplementary_Data [file msad110_supplementary_data.zip › Supplementary_figures.pdf]

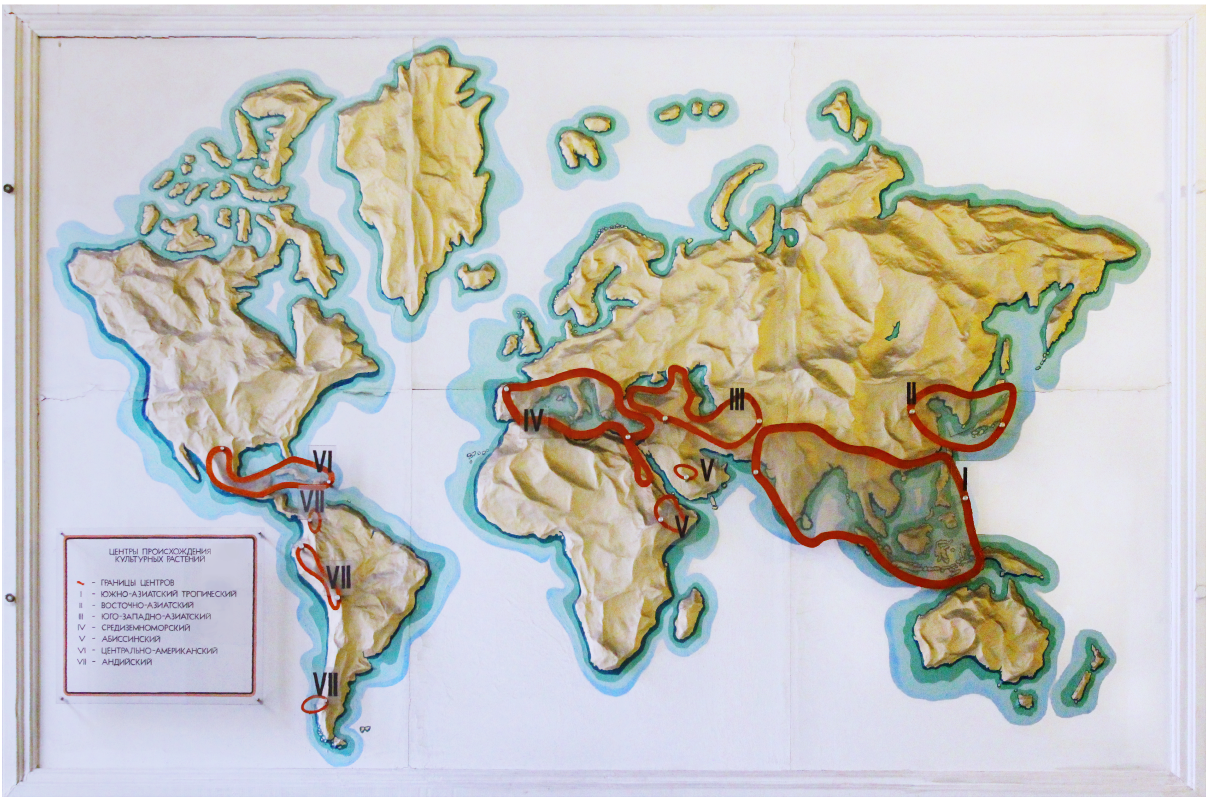

Supplementary Figure S1. Vavilov's centers of domestication (outlined in red) and our hypothesized paths of the desi spread shown as yellow lines (some of which are known and some are tested). The map is from the Vavilov Institute of Plant Genetic Resources (Photo: A.A. Igolkina)

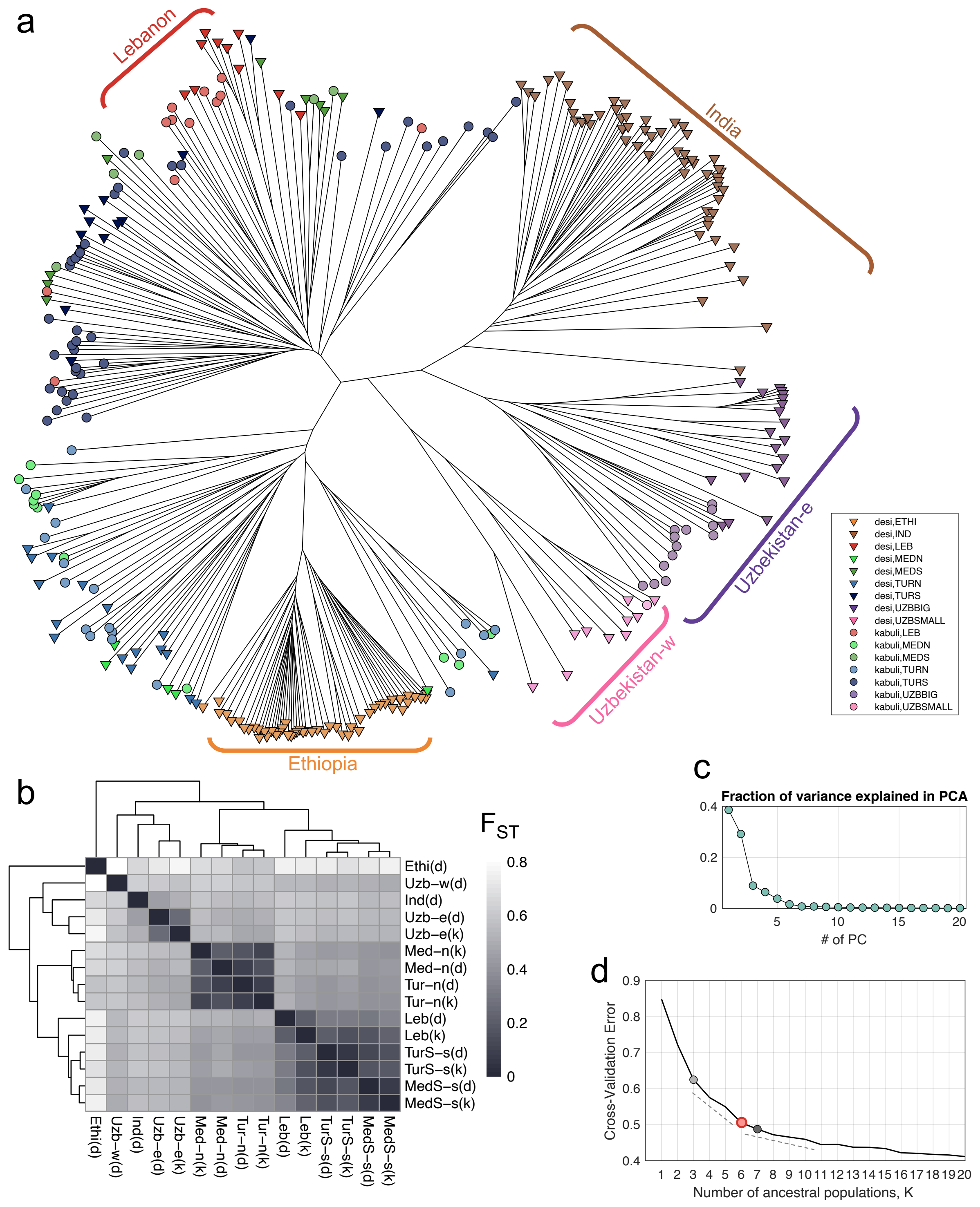

Supplementary Figure S2. Population structure of chickpea landraces.

(a) Neighbor-joining tree of chickpea accessions using SNP-distance. The ten chickpea subpopulations are marked with different colors. (b) Mean pairwise F_{ST} comparison of chickpea populations.

(c) proportion of variance explained by PCs in PCA analysis on all SNP data.

(d) Cross-validation plot for different numbers of ancestral populations used in the ADMIXTURE program. The curve does not show a minimum, that is a criterion for K choice. K is the number

of subpopulations that make up the total population. Two points reflect cross-validation errors for runs demonstrated below.

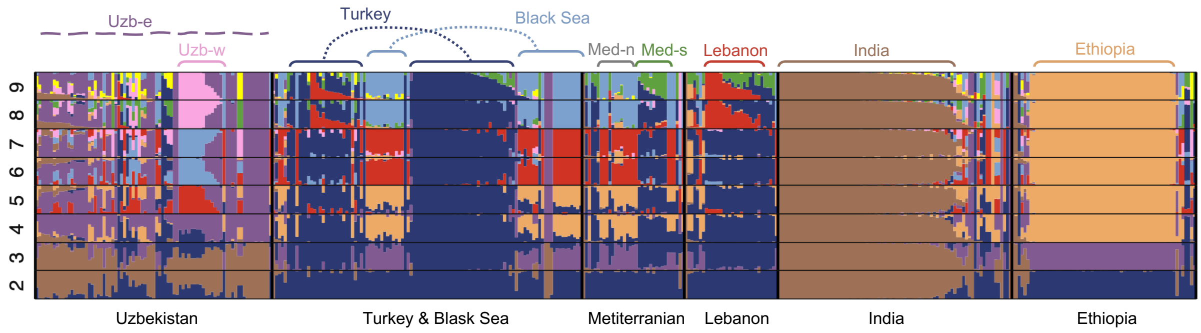

Supplementary Figure S3. Population structure for the unfiltered chickpea dataset inferred using ADMIXTURE analysis. ADMIXTURE results at K = 2..9 are shown. Each accession is represented by a vertical stacked bar indicating the proportions of ancestry in K predicted ancestral populations. Y axis represents different K. Vertical borders separate geographical regions; names of the geographical regions are at the bottom. The approximate position of the accessions after filtration is shown with brackets at the top.

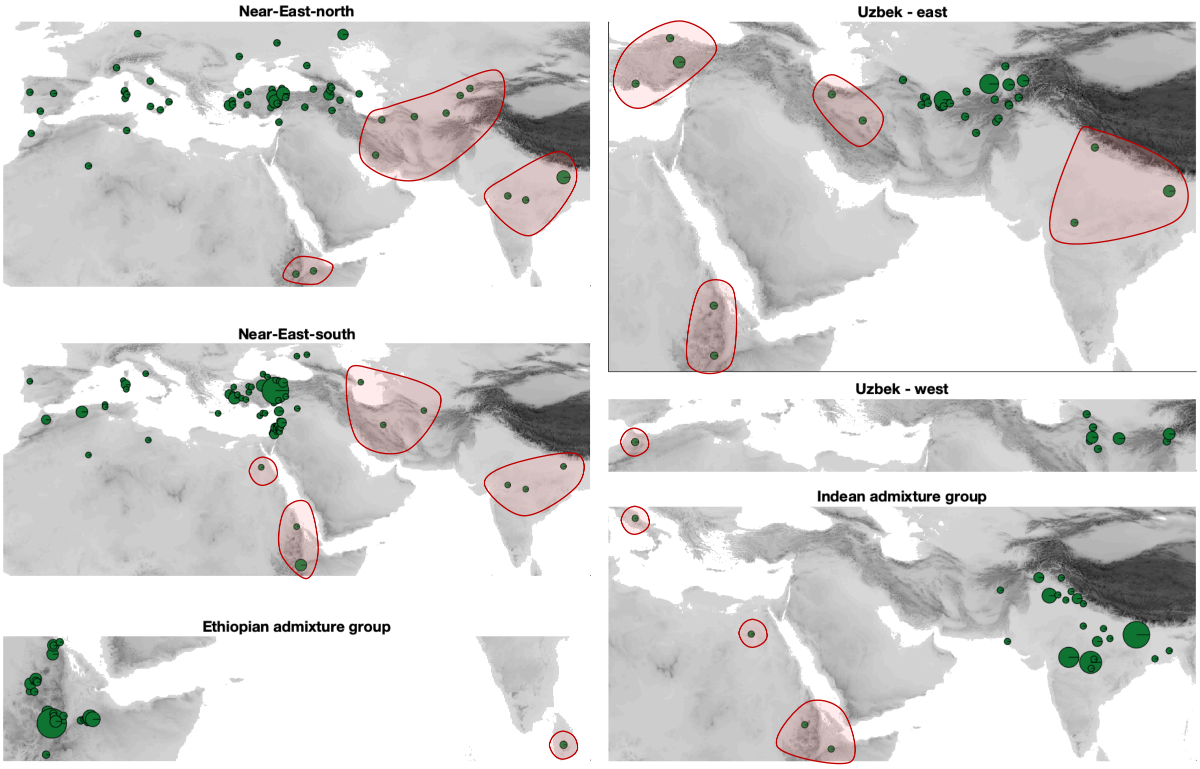

Supplementary Figure S4. Sampling sites of six admixture groups. Read areas denote samples which have the discrepancy between genetic group and geographical rehion.

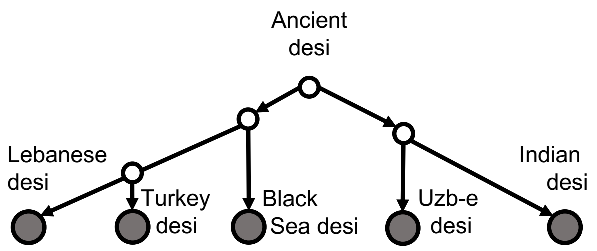

Supplementary Figure S5. Known topology of the chickpea desis based on historical and archeological evidence.

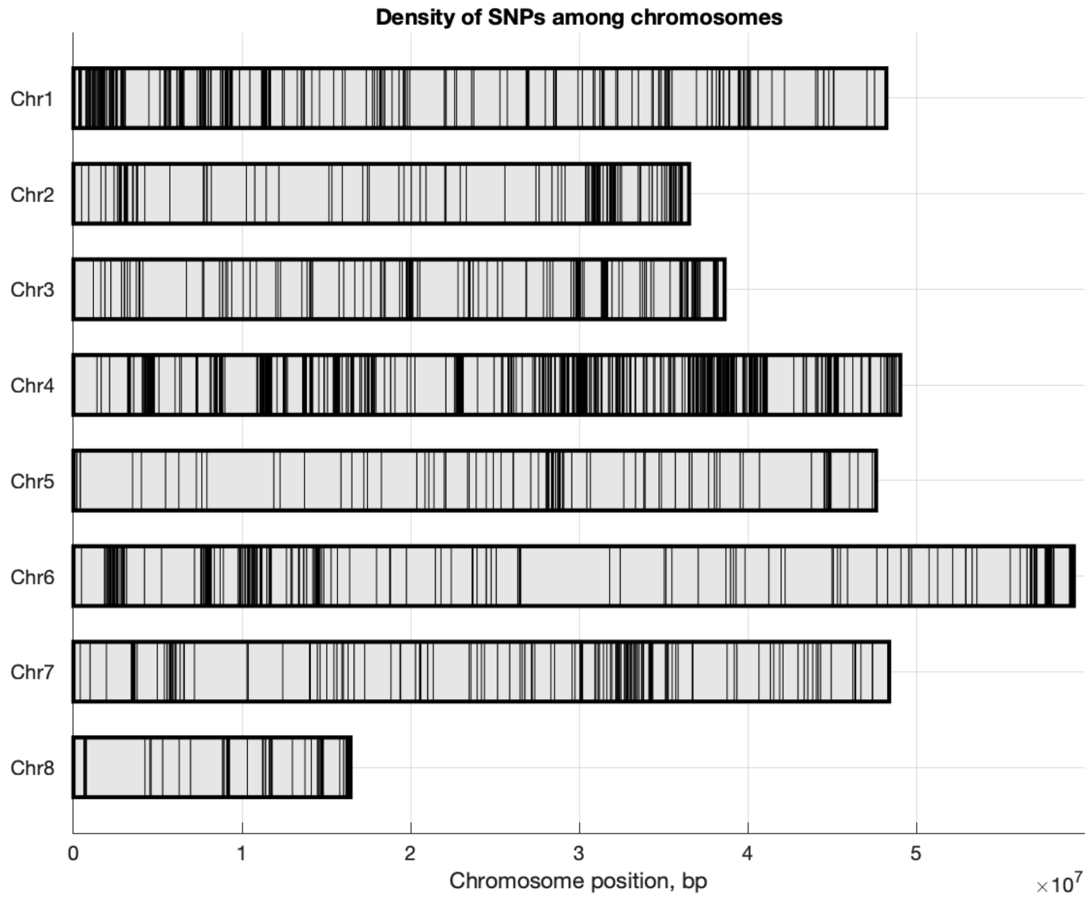

Supplementary Figure S6. Density of SNPs along the chromosomes. Each vertical line corresponds to the position of one SNP.
